# Cell Density and mRNA Expression of Inhibitory Interneurons in Schizophrenia: A Meta-Analysis

**DOI:** 10.1101/2025.05.23.655812

**Published:** 2025-05-27

**Authors:** Aidan G. Mulvey, Kaitlyn M. Gabhart, Tineke Grent-’t-Jong, Suzana Herculano-Houzel, Peter J. Uhlhaas, André M. Bastos

**Affiliations:** 1 Vanderbilt University, Department of Psychology, Nashville, TN; 2 Department of Child and Adolescent Psychiatry, Charité-Universitätsmedizin, Berlin

## Abstract

**Importance:**

GABAergic inhibitory interneurons have been implicated in the pathophysiology of schizophrenia. However, there is conflicting evidence regarding the nature and extent of the deficits across brain areas and interneuron subtypes.

**Objective:**

The primary objective was to determine the extent of changes in parvalbumin, somatostatin, calbindin, and calretinin interneurons across brain regions and cortical layers in schizophrenia compared to healthy controls. The secondary objective was to examine differences in neuronal density and neuronal mRNA expression of GABAergic interneurons.

**Data Sources:**

A comprehensive search was conducted from Fall 2024 to Spring 2025, including terms related to schizophrenia, interneurons, immunohistochemistry and mRNA. Only post-mortem human studies containing neuroanatomical data of parvalbumin, somatostatin, calbindin, and calretinin interneurons were considered.

**Study Selection:**

We selected immunohistochemistry and mRNA studies that examined parvalbumin, somatostatin, calbindin, and calretinin interneuron density or expression in schizophrenia patients.

**Data Extraction and Synthesis:**

Data were extracted following PRISMA guidelines and were pooled with a random-effects model. A t-score statistic was applied to obtain distributions of effects. A two-sample t-test and Cohen’s effect size was used to compare across areas, layers, cell types and methods.

**Main Outcome(s) and Measure(s):**

Primary measurements/outcome were laminar interneuron density (assessed by immunohistochemistry) and gene expression (assessed by mRNA).

**Results:**

Data from 28 immunohistochemistry studies (362 control participants, 335 individuals with schizophrenia) revealed that the hippocampus and prefrontal cortex were most consistently characterized by alterations in GABAergic interneurons; parvalbumin and somatostatin interneuron density was reduced in the hippocampus, while data from 18 mRNA studies (524 control participants, 519 individuals with schizophrenia) indicated reduced parvalbumin and somatostatin expression in prefrontal cortex. Layer-specific analysis demonstrated that parvalbumin interneurons were most affected in the superficial layers of prefrontal cortex, while somatostatin interneurons exhibited the strongest deficits in layers 2 and 5.

**Conclusions and Relevance:**

Our results show that GABAergic interneurons in the PFC and hippocampus are particularly affected. Parvalbumin and somatostatin showed the largest deficits, involving superficial layers and layer 5. We also identified significant reductions in parvalbumin and calretinin interneuron density in subcortical areas. Together, these data have important implications for the pathophysiology and computational models of circuits deficits in the disorder.

## INTRODUCTION

Schizophrenia (ScZ) is a severe psychiatric disorder characterized by positive symptoms, such as delusions and hallucinations, and negative symptoms including anhedonia and reduced affect.^1^ In addition, individuals with ScZ have pronounced cognitive deficits involving basic sensory processes as well as dysfunctions in higher cognitive processes.^2,3^

Despite significant advances in understanding the pathophysiology of the disorder,^4,5^ the underlying mechanisms and localization of circuit deficits remain unclear. Thus, different cortical and subcortical areas, including the prefrontal cortex (PFC),^5^ hippocampus,^6^ and thalamus^7^, and in addition aberrant neurotransmission with dopamine,^8^ glutamate^9^ and gamma-aminobutyric acid (GABAergic)^4,10^ have been proposed in circuit models of ScZ.

Converging evidence from post-mortem studies suggests that GABAergic interneurons are impaired in ScZ.^5,11,12^ GABAergic interneurons are a fundamental aspect of neural circuits due to their role in exerting precise rhythmic inhibition of pyramidal cells thereby leading to the emergence of neural oscillations.^13,14^ Different subtypes of inhibitory interneurons with distinct morphological and functional characteristics can be distinguished based on their expression of calcium-binding proteins: 1) Parvalbumin (PV) 2) Calbindin (CB) and 3) Calretinin (CR) inhibitory interneurons, as well as based on transcriptomic factors: 1) Somatostatin (SST), 2) vasoactive intestinal peptide, 3) neuropeptide Y, 4) cholecystokinin, and 5) 5-HT3A receptors.^12^

Studies using immunohistochemistry (IHC) report deficits in cell density of GABAergic interneurons in ScZ.^15–17^ In addition, mRNA expression reductions of interneuron subtypes have been reported across cortical and subcortical brain regions.^5,11,18–20^ However, a comprehensive overview of deficits in GABAergic interneurons, the brain regions, and layers involved is currently not available. Previous studies have focused on PV interneurons in the PFC in ScZ,^11,21–23^ reporting reductions of mRNA expression in layers 2, 3, and 4. However, these results are not consistent,^17^ and evidence suggests these alterations extend to other cortical areas,^24,25^ as well as to subcortical and hippocampal regions.^26,27^

We conducted a meta-analysis of human post-mortem ScZ studies to examine changes in PV, CB, CR and SST interneurons across hippocampal, cortical, and subcortical regions. We focused on PV, CB, CR, and SST neurons as the link between the other cell types and ScZ is weaker.^12,28^ Specifically, data from IHC and mRNA studies were analyzed to determine whether deficits in ScZ involved reduced neuronal density (quantified by IHC studies) and/or altered mRNA expression levels of GABAergic interneurons.

## METHODS

### Search strategy and selection criteria

Following PRISMA guidelines,^29^ ProQuest, PubMed, and Google Scholar were searched with the following search terms: ‘schizophrenia OR ScZ OR SZ AND interneurons’, ‘(interneuron density) AND schizophrenia, ‘immunohistochemistry AND schizophrenia, ‘(layer-specific cell density) AND schizophrenia, ‘(interneuron density) AND (clinical disorders)’, ‘Parvalbumin OR Calbindin OR Calretinin OR Somatostatin AND schizophrenia, ‘(layer-specific interneuron deficit) AND schizophrenia, ‘(mRNA expression) AND schizophrenia, ‘(interneuron mRNA) AND schizophrenia’, and ‘(interneuron expression) AND schizophrenia’.

**Figure 1.**
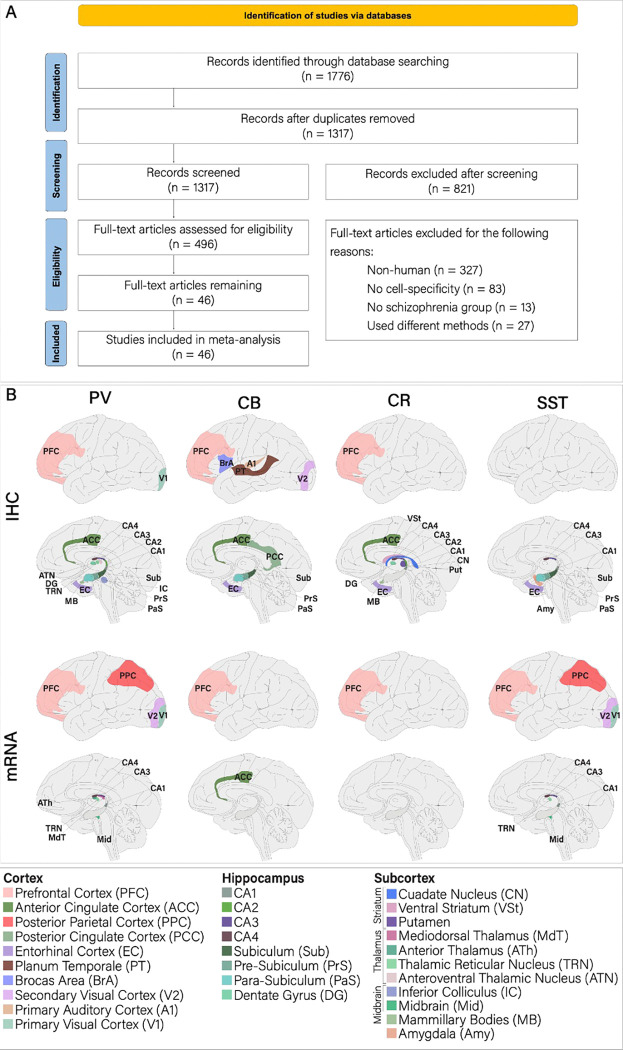
A) PRISMA 2020 flowchart for study screening and inclusion. B) Each brain region that a record analyzed (mRNA and IHC) is shaded in its own designated color. Abbreviations used throughout the present work are given by the Figure 1B legend.

We identified a total of 496 studies after duplicate removal and title screening of which 450 studies were excluded for the following reasons (Figure 1A): 1) 327 studies of non-human samples, 2) 83 studies did not include cell-specific data, 3) 13 studies did not include ScZ patients, 4) 27 studies had different methodologies. The final sample included 46 studies that accumulated a total of 827 healthy controls (HCs) and 800 individuals with ScZ. The asterisk (*) in the columns containing sample sizes shows studies that used the same samples. However, we treated the samples as independent because each sample analyzed different brain regions or cell types (Table 1).

A quantitative meta-analysis was performed on IHC studies (*n* = 28), where we examined the interneuron density for PV (*n* = 21), CB (*n* = 9), CR (*n* = 11), and SST (*n* = 4) studies. Similarly, we included 19 studies, including one study with both IHC and mRNA data,^27^ examining mRNA expression levels for PV (*n* = 14), CB (*n* = 2), CR (*n* = 4), and SST (*n* = 14) studies.

### Data extraction

The laminar mean cell densities and standard error of means (SEMs) were obtained for each brain area using WebPlotDigitizer,^30^ unless the exact means and SEMs were explicitly reported in the manuscript. The SEM and mean density were derived from each layer in each brain region to quantify PV, CB, CR, and SST interneuron densities and mRNA expression.

Included studies quantified mRNA expression using grain analysis, optical density analysis, fluorescent in situ hybridization (FISH), or quantitative real-time PCR (qPCR). Importantly, no statistical differences have been identified between FISH and qPCR, which we confirmed in our data set with a two-sample t-test (PV: *p* = 0.20, SST: *p* = 0.60).^31^

### Data synthesis and statistical analysis

We applied a t-score framework (equation 1), which allows comparison across studies by standardizing the mean differences to their uncertainty values and eliminating any variation in units.

(equation 1) $$t^{\prime }=\frac{a-b}{\sqrt{u(a{)}^{2}+u(b{)}^{2}}},$$

in which a and b are the mean densities/expressions in ScZ and HC, respectively, and u(a) and u(b) are the SEMs of ScZ and HC, respectively, across individuals within a study. Thus, a negative t-score suggests a cellular or transcriptional deficit in ScZ, and a positive t-score suggests a cellular or transcriptional deficit in HC. The t-score was derived from each cortical layer in each study. If a study combined layers (i.e. L5/6), the resulting t-score was used for both layers.

23 studies reported whole-area data, instead of laminar-specific results. For such studies, a single t-score was calculated from the mean and SEM of each area. To best compare effects across areas, a mean t-score was calculated for each laminar study by combining the laminar means and SEMs into a single mean and SEM for that study for both ScZ and HC, followed by a t-score. To assess significance, we report the t-score with the p-value of a one-sample t-test.

**Figure 2.**
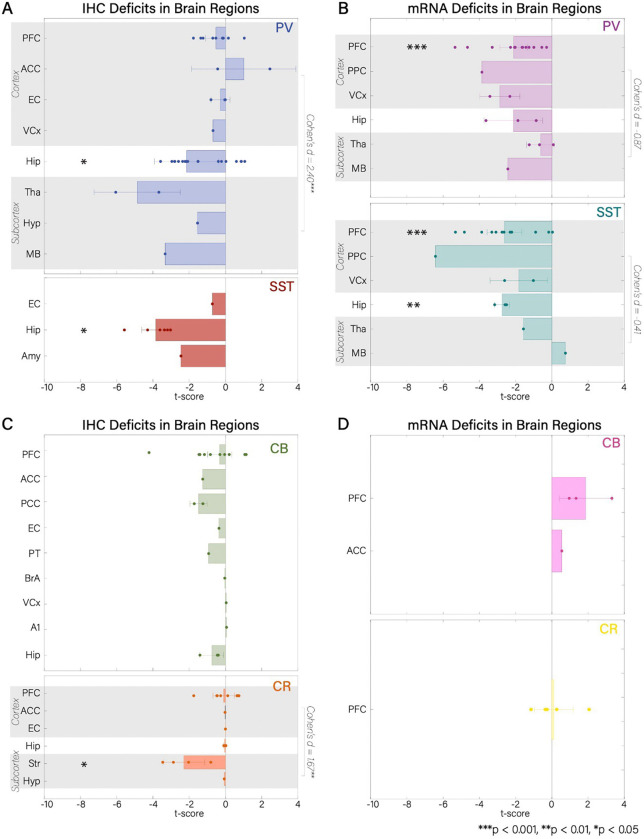
t-scores for IHC and mRNA methods in all brain regions and cell types that were included in the analysis. Negative t-scores reflect deficits in ScZ. Data points are individual studies. Gray shadings are demarcations of areas included in either cortex or subcortex for effect size analyses (Cohen’s d included). A) Comparison of IHC t-scores for PV and SST interneurons. B) Comparison of mRNA t-scores for PV and SST interneurons. C) Comparison of IHC t-scores for CB and CR interneurons. D) Comparison of mRNA expression t-scores for CB and CR interneurons.

PFC was frequently subdivided into Brodmann areas 9, 10, 11, and 46, which are collectively referred to as PFC for the present meta-analysis. Additionally, we will broadly refer to different brain structures as such cortex (PFC, ACC, PPC, PCC, EC, PT, BrA, V2, A1, V1), hippocampus (CA1–4, Sub, PrS, PaS, and DG), thalamus (MdT, ATh, TRN, and ATN), striatum (VSt and Put), midbrain (IC and Mid), and subcortex (thalamus, striatum, midbrain, amygdala [Amy], and hypothalamus [MB]; Figure 1B). Thus, if a study quantified multiple structures within a region (i.e. CA1 and CA2), a t-score was taken for each structure (Figure 2C), but grouped for regional analysis (Figure 2A–B). To compare extent of effects, we used a two-sample t-test and Cohen’s effect size analysis on the t-scores for each region.

### Assessing publication bias and mediating factors

Publication bias was assessed using visual inspection of funnel plots and Egger’s regression testing for all areas and studies for each cell type and method, where n>5. To calculate standard error of the effect, we used equation 2, where N is the number of subjects from the ScZ group (A) control group (B) and $t^{’}$ is the t-score of the study. The standardized effect size was regressed against the inverse of the standard error (equation 2) and the intercept of this regression $\left(B_{0}\right)$ was then assessed for evidence of publication bias.^32^

(equation 2) $$SE\left(t^{\prime }\right)=\sqrt{\frac{N_{A}-N_{B}}{N_{A}N_{B}}+\frac{{t\prime }^{2}}{2\left(N_{A}+N_{B}\right)}}$$

We also performed a one-sample t-test across the data set to determine significant effects of the t-score (Figure 4). For biased sets, we removed outlier data points until $p\left(B_{0}\right)\geq 0.05$.. We conducted a one-sample t-score testing with a minimum of 5 data points at all times. After removal, we reassessed significance with a one-sample t-test to determine if the bias affected the significance.

We extracted demographic data, where possible, from each study (Table 1). To assess confounds, we used regression models of t-score and mean age, male-female ratio, post-mortem interval (PMI), and brain pH. For all factors, we took a weighted average of HC and ScZ parameters to get a single value per model input. We report the regression coefficient $\left(B_{1}\right)$ and the Bonnferroni-corrected p-value $\left(p\left(B_{1}\right)\right)$ of each regression (see Supplemental Fig. 4).

## RESULTS

## Alterations of inhibitory interneurons per brain area in IHC and mRNA studies

When considering areas as a whole and combining sub-area designations and layers, the hippocampus and the PFC were the only areas with significant alterations.^a^ The density of PV (*t*′ = −2.14, *df* = 18, *p* < 0.05) and SST (*t*′ = −3.30, *df* = 5, *p* < 0.001) interneurons was decreased in the hippocampus (Figure 2A), as well as mRNA expression for SST (*t*′ = −2.75, *df* = 2, *p* < 0.01; Figure 2B). The density of PV (*t*′ = −2.14, *df* = 18, *p* < 0.05) and SST (*t*′ = −3.30, *df* = 5, *p* < 0.001) interneurons was decreased in the hippocampus (Figure 2A), as well as mRNA expression for SST (*t*′ = −2.75, *df* = 2, *p* < 0.01; Figure 2B). While mRNA expression for both PV (*t*′ = −2.11, *df* = 13, *p* < 0.001; Figure 2B) and SST (*t*′ = −2.62, *df* = 11, *p* < 0.001) interneurons was reduced in PFC, neuronal density was not reduced for CB (*t*′ = −0.32, *df* = 8,*p* < 0.05), CR (*t*′ = −0.10, *df* = 7, *p* < 0.05), nor PV (*t*′ = −0.52, *df* = 9, *p* < 0.05; Figure 2A, C).

We found reduced density in subcortical structures (PV: Tha, MB; CR: Str, Hyp) for PV (*t*′ = −3.65, *df* = 3, *p* < 0.05) and CR (*t*′ = −1.85, *df* = 4, *p* < 0.05) interneurons. Compared to cortical areas (PV: PFC, ACC, EC, V1; CR: PFC, ACC, EC), deficits in subcortical regions were significantly larger (PV: *t*(17) = 4.95, *p* < 0.001, *Cohen’s d* = 2.40; CR: *t*(13) = 3.25, *p* < 0.01, *Cohen’s d* = 1.67; Figure 2A, C; Figure 4A; Supplemental Fig. 3A). mRNA expression studies showed a trend in the opposite direction, with slightly larger effects in cortex compared to subcortex for PV (*t*(19) = −1.64, *p* = 0.12, *Cohen’s d* = −0.87; cortex: PFC, PPC, V1, V2; subcortex: Tha, MB; Figure 1B; Figure 4B; Supplemental Fig. 3A) and for SST interneurons (*t*(15) = −0.57, *p* = 0.58, *Cohen’s d* = −0.41; cortex: PFC, PPC, V1, V2; subcortex: Tha, MB; Figure 1B; Figure 4C; Supplemental Fig. 3A).

We also found significant reductions when taking the mean t-score across all brain regions for cell density of PV (*t*′ = −1.57, *df* = 37, *p* < 0.05; Figure 4A), CB (*t*′ = −0.53, *df* = 19, *p* < 0.05; Figure 4B), and SST (*t*′ = −2.82, *df* = 7, *p* < 0.001; Figure 4B) and mRNA expression of PV (*t*′ = −2.08, *df* = 23, *p* < 0.001; Figure 4C) and SST (*t*′ = −2.53, *df* = 19, *p* < 0.001; Figure 4D), but not for CR density (Figure 4B) and CB and CR mRNA expression.

### Layer-resolved alterations of inhibitory interneurons in cortex

**Figure 3.**
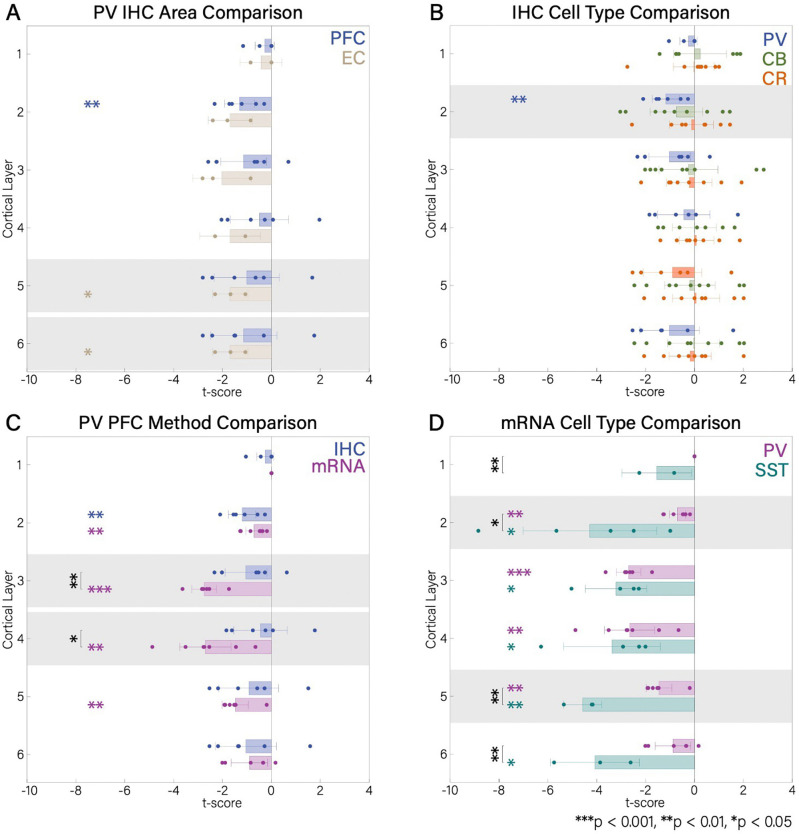
Laminar distributions and comparisons of data points in A) PFC vs. EC in IHC, B) cell type (PV, CB, CR) IHC, C) PV PFC IHC vs. mRNA, and D) PV and SST mRNA. +/− 2 SEM is given in error bars. Colored asterisks represent significance at 3 different levels (*** P<0.001, ** P<0.01, *P<0.05) on a one-sample t-test to assess brain areas/cell types that are affected (different between ScZ and HC). Black asterisks with bars represent significance at 3 levels (*** P<0.001, ** P<0.01, *P<0.05) comparing the indicated distributions, with a two-sample t-test.

**Figure 4.**
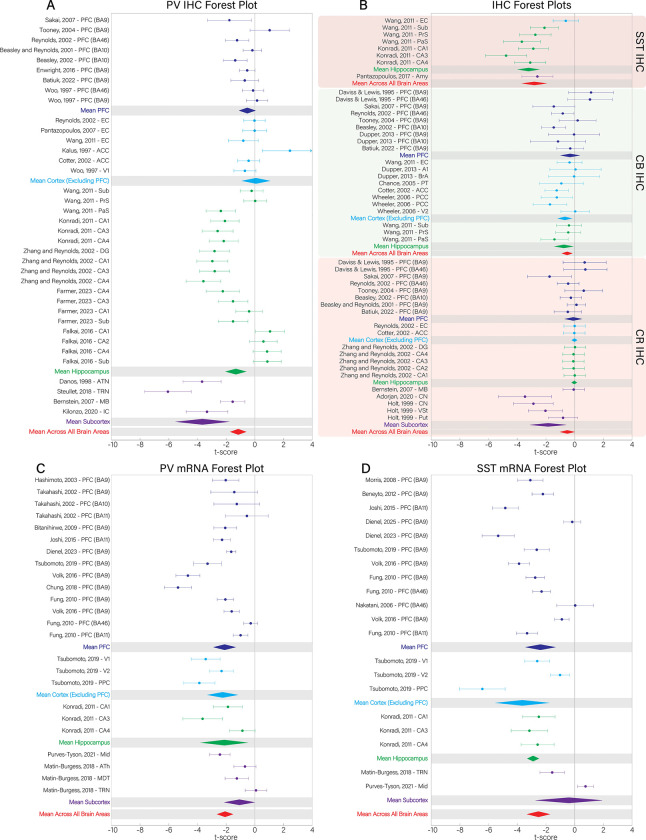
Forest plots of all studies for A) PV IHC, B) SST, CB, and CR IHC, C) PV mRNA, and D) SST mRNA data sets. Dots are the t-score ± 1 standard error of the effect. Regional mean t-scores ± 2*SEM are plotted as diamonds. The mean across all brain areas ± 2*SEM is plotted in the red diamond. T-score of area CA2 from Zhang & Reynolds was removed from subpanel A (t = −16.918 ± 4.54), because it is a large outlier.

Although PFC interneuron density was not affected when all layers were combined (Figures 2, 4), PV interneuron density was decreased in L2 (*t*′ = −1.17, *df* = 5, *p* < 0.01; Figure 3B), but not in other layers. In addition, the EC showed a specific reduction in PV interneuron density in L5/6 (*t*′ = −1.52, *df* = 2, *p* < 0.05; Figure 3A). CB and CR interneurons were intact, both at the whole area and layer-specific resolution (*p* > 0.05; Figure 3B).

Reductions in mRNA expression were more widespread across layers. PV mRNA expression was decreased in PFC in L2-L5, with the strongest effect observed in L3 (L2: *t*′ = −0.70, *df* = 6, *p* < 0.01; L3: *t*′ = −2.80, *df* = 5, *p* < 0.001; L4: *t*′ = −2.65, *df* = 6, *p* < 0.01; L5: *t*′ = −1.45, *df* = 5, *p* < 0.01; Figure 3C). SST interneuron mRNA expression was reduced in L2–6 in PFC, with the strongest deficit in L5 (L2: *t*′ = −4.28, *df* = 4, *p* < 0.05; L3: *t*′ = −3.21, *df* = 3, *p* < 0.05; L4: *t*′ = −3.37, *df* = 3, *p* < 0.05; L5: *t*′ = −4.58, *df* = 2, *p* < 0.01; L6: *t*′ = −4.08, *df* = 2, *p* < 0.05). Notably, deficits in SST expression were statistically larger than PV expression deficits in L1, 2, 5, and 6 (all comparisons, *p* < 0.01, Figure 3D).

### Comparison between IHC and mRNA Expression Studies

mRNA expression of PV interneurons was more affected than PV interneuron density in PFC (*t*(21) = 2.96, *p* < 0.01, *Cohen’s d* = 1.22; Supplemental Fig. 3B), particularly in L3 and L4 (*p* < 0.01 and *p* < 0.05, respectively; Figure 3C, Supplemental Fig. 3C). In contrast, neuronal density was more affected than mRNA expression in subcortical (IHC: Tha, Hyp, MB; mRNA: Tha, MB) PV interneurons (*t*(5) = −3.45, *p* < 0.05, *Cohen’s d* = −2.21; Supplemental Fig. 3A). However, deficits in mRNA expression and neuronal density were comparable in hippocampal PV interneurons (*t*(20) = −0.01, *p* = 0.99, *Cohen’s d* = −0.005; Supplemental Fig. 3B) and SST interneurons (*t*(7) = −1.09, *p* = 0.31, *Cohen’s d* = −0.68; Supplemental Fig. 3B).

### Publication bias and mediating factors

Our analysis of publication bias using funnel plots and Egger’s test determined that data sets for PV (*B*_0_ = −6.62, *df* = 36, *p* < 0.001), CR (*B*_0_ = −3.69, *df* = 19, *p* < 0.05), and SST (*B*_0_ = −14.62, *df* = 6, *p* < 0.001) density exhibited evidence of publication bias (Supplemental Fig. 5). CB interneuron density was unbiased (*B*_0_ = −2.01, *df* = 18, *p* > 0.05). To correct for this identified bias, we removed 4 and 1 data points for PV and CR, respectively (circled dots in Supplemental Fig. 5). SST remained biased even after significant removal of data points. When we reassessed significance, reductions in PV interneuron density remained significant (*t*′ = −0.86, *df* = 33, *p* < 0.001). For mRNA, PV (*B*_0_ = −3.12, *df* = 22, *p* < 0.05) and CR (*B*_0_ = −2.43, *df* = 3, *p* < 0.05) data sets were unbiased. The SST data set was biased (*B*_0_ = −9.41, *df* = 18, *p* < 0.001). SST mRNA expression deficits remained significant after removing 4 studies (*t*′ = −1.88, *df* = 14, *p* < 0.001). There were data points (fewer than 5 data points) to perform this analysis on the CB mRNA expression data set.

We also tested for whether the reported effects could have been affected by age, sex, brain pH, and post-mortem interval (PMI). After Bonnferroni correction (*n* = 28 regressions), we found no significant correlations between these factors and PV IHC, CB IHC, SST IHC, PV mRNA, CR mRNA, and SST mRNA (regressions, all *p* > 0.0018, Supplemental Fig. 4). The regressions of mediating factors revealed significant relationships between CR interneuron density t-score with male-female-ratio (*B*_1_ = −2.49, *p* < 0.0018) and brain pH (*B*_1_ = −21.50, *p* < 0.0018) (Supplemental Fig. 4B, C, E, F). There was insufficient data for all regressors of CB mRNA expression, as well as for brain pH and SST interneuron density (fewer than 5 studies).

Finally, it is important to consider medication status. Of the 46 studies in our meta-analysis, 9 reported data on Chlorpromazine (CPZ) equivalent doses. All studies overlapped in their reported ranges of CPZ-doses (Supplemental Fig. 2).

## DISCUSSION

These data provide a comprehensive meta-analysis of changes in GABAergic interneurons from post-mortem data in ScZ. Our results show that alterations in GABAergic interneurons were most consistently observed in the hippocampus and PFC with PV and SST interneurons particularly affected in ScZ. While CB cell density was overall reduced, CR was mostly intact. Layer-specific analyses of PFC revealed PV deficits were most pronounced in superficial L2/3, while SST interneurons exhibited the strongest deficits in superficial L2 and deep L5.

### GABAergic Interneurons and Brain Areas in ScZ

Current theories have implicated different cortical and subcortical brain regions in ScZ with deficits in GABAergic interneurons reported across frontal^17^ and sensory areas,^33–36^ hippocampus,^27,37–39^ as well as the basal ganglia^40^ and the limbic system.^10^ The current findings highlight that GABAergic interneurons in the hippocampus and PFC are particularly affected. Circuit deficits in the PFC have been implicated in a range of cognitive deficits in ScZ, in particular working memory^41^ and executive functions.^42,43^ Impaired hippocampal functioning has been proposed as an early signature of psychosis^44,45^ and could also be related to reduced functionality of the PFC,^46^ and impaired hippocampal–prefrontal connectivity.^47^

### GABAergic Interneuron Subtypes and Layer-Specific Deficits

Among the subtypes of GABAergic interneurons, PV and SST were particularly affected in ScZ, which is consistent with genomics and transcriptomics work that have identified risk genes associated with these cell types in ScZ.^48,49^ In addition, there is consistent evidence of PV deficits in animal models of ScZ in PFC and hippocampus.^50^

PV interneurons are involved in the generation of gamma-band (30–100 Hz) oscillations,^51–53^ which are impaired in ScZ (for a review, see Uhlhaas & Singer, 2010).^54^ SST interneurons have also been associated with the generation of gamma-band oscillations^51,53,55^ but also with slower frequencies, including theta band (4–6 Hz)^56,57^ and beta band (15–30 Hz)^58^ oscillations.

We found significant reductions in PV and SST mRNA expression across cortical layers in PFC. PV mRNA expression was most reduced in L3/4, where this cell type is highly enriched.^59^ The mRNA deficits of SST interneurons span those of PV, with the largest deficits in L2 and deep layers 5/6. These layer-specific deficits, paired with the deficits uncovered at the whole-area resolution, have implications for computational models of ScZ.

One class of influential models is the predictive coding approach.^8,60,61^ According to these models, prediction errors are computed by L2/3 pyramidal neurons,^62^ which are under the inhibitory control of local interneurons in these layers. Deficits in prediction errors in ScZ could underlie abberant sensory inferences, leading to delusions and hallucinations.^61^ In addition, gamma-band oscillations in superficial layers (L2/3) support feedforward prediction error propagation, while deep layers (L5/6) are associated with lower-frequency alpha (8–12 Hz)/beta oscillations, often implicated in feedback processing, top-down control, and long-range communication.^58,59,62–66^ Together these data suggest that impaired GABAergic interneurons in ScZ could lead to impaired feedforward processing (and reduced prediction error) as well as reduced top-down feedback which could contribute towards hallucinations and delusions and possibly also cognitive deficits.

### Conceptualizing Differences Between IHC and mRNA Results

Our results from IHC and mRNA studies provided converging but also distinct perspectives on GABAergic interneuron deficits in ScZ. Interneuron density in subcortical structures was more reduced than in cortical regions for PV and CR interneurons. We found the opposite trend in mRNA expression of PV and SST interneurons. It should also be noted that ScZ may be characterized primarily by loss of mRNA without loss of neurons in many structures.^11^ Therefore, it is possible that subcortical PV and CR interneurons are more vulnerable to aberrant developmental processes in ScZ compared to their cortical counterparts. Future studies could examine the distinctions between these cortical and subcortical PV/CR cells in greater detail.

### Limitations

Several limitations warrant consideration when interpreting this meta-analysis. Firstly, the included studies exhibited unequal sampling across different brain regions, cell types, and cortical layers. This could be addressed in future studies with a broader and more equal sampling across the brain. Second, these results cannot definitively determine whether the observed PV and SST interneuron deficits represent a primary pathology in ScZ or arise secondary to other factors, such as impairments in excitatory neurotransmission (for a review, see Dienel et al., 2022).^67^

### Summary

This meta-analysis, which encompassed over 1,500 participants, 29 brain regions, and 4 inhibitory cell types, provides a comprehensive overview of GABAergic interneuron deficits in ScZ from post-mortem data, with implications for the pathophysiology of the disorder. The convergence on PV and SST interneuron deficits in PFC and hippocampal circuits is important for current computational and circuit models.^57,68^ Future investigations should also explore the functional consequences of layer-specific deficits and their contribution to cognitive impairments in ScZ as well as the relationship to other aspect of circuit deficits, such as impaired excitatory neurotransmission.^69,70^ Additionally, these results could guide the development of targeted interventions aimed at restoring the function of PV and SST interneurons through pharmacological^71^ or neuromodulatory^72^ approaches.

## Supplementary Material

Supplement 1

Supplement 2

## Figures and Tables

**Table 1.** Quantitative data from all included studies that investigated the density or expression of interneuron markers in HC vs. ScZ.

Author	Year	Method	Area	Cell Type	N HC	N SCZ	Laminar
Density							
Sakai	2007	IHC	BA 9	PV, CB, CR	5	7	Y
Tooney	2004	IHC	BA 9	PV, CB, CR	6	6	Y
Reynolds	2002	IHC	BA 46	PV, CB, CR	15	15	Y
			BA 28	PV, CR			
Beasley and Reynolds	1997	IHC	BA 10	PV	15*	15*	Y
Beasley	2002	IHC	BA 9	PV, CB, CR	15*	15*	Y
Beasley and Reynolds	2001	IHC	BA 9	PV, CR	22	18	Y
Enwright	2016	IHC	BA 9	PV	28	28	Y (L3)
Batiuk	2022	IHC	BA 9	PV, CR	10*	10*	Y
				CB	6*	6*	Y
Pantazopoulos	2007	IHC	BA 28	PV	16	10	N
Woo	1997	IHC	BA 17, 9, 46	PV	15	15	N
Kalus	1997	IHC	BA 24	PV	5	5	Y
Daviss & Lewis	1995	IHC	BA 9, 46	CB, CR	5	5	Y
Dupper	2013	IHC	BA 9, 46, 41, 44	CB	5	3	Y
Chance	2005	IHC	BA 22	CB	12	12	Y
Wang	2011	IHC	BA 28	PV, SST, CB	17	11	Y
			Sub, PrS, PaS	PV, SST, CB			N
Wheeler	2006	IHC	BA 23, 30, 18	CB	9	9	N
Cotter	2002	IHC	BA 24	PV, CB, CR	15	15	Y
Danos	1998	IHC	ATN	PV	14	12	N
Steullet	2018	IHC	TRN	PV	20	15	N
Berstein	2007	IHC	MB	PV	15	15	N
Konradi	2011	IHC	CA 1, 2/3, 4	SST	20*	12*	N
		IHC		PV	18*	10*	N
Zhang and Reynolds	2002	IHC	DG, CA1–4	PV, CR	15	15	N
Farmer	2023	IHC	CA1–4, Sub	PV	11	10	N
Falkai	2016	IHC	CA1, 2/3, 4, Sub	PV	10	10	N
Kilonzo	2020	IHC	IC	PV	20	20	N
Adorjan	2020	IHC	CN	CR	6	6	N
Holt	1999	IHC	CN, VStr, Put	CR	9	10	N
Pantazopoulos	2017	IHC	Amygdala	SST	15	12	N
mRNA							
Dienel	2025	fluorescence in situ hybridization (FISH)	BA 9	SST	46	46	Y
Bitanihirwe	2009	FISH	BA 9	PV	20	20	Y
Dienel	2023	in situ hybridization	BA 9	PV, SST	29	29	Y
Beneyto	2012	in situ hybridization	BA 9	SST (R1, R2)	23	23	Y
Joshi	2015	In situ hybridization, quantitative PCR	BA 11	PV, SST	38	38	Y
Morris	2008	in situ hybridization	BA 9	SST	23	23	Y
Takahashi	2002	in situ hybridization	BA 9, 10, 11	PV	5	6	Y
Hashimoto	2003	film optical density	BA 9	PV, CR	15	15	Y
Fung	2010	quantitative RT-PCR	BA 9	PV, SST, CB, CR	37	37	N
Nakatani	2006	quantitative RT-PCR	BA 46	SST	7	7	N
Volk	2016	quantitative PCR	BA 9	PV, SST	62	62	N
Purves-Tyson	2021	quantitative PCR	Midbrain	PV, SST	28	28	N
Matin Burgess	2018	in situ hybridization	Thalamus	PV, SST	14	14	N
Fung	2014	quantitative PCR	BA 46, 11	PV, CB, CR, SST	35	35	N
Chung	2018	quantitative PCR	BA 9	PV	40	40	N
Tsubomoto	2019	quantitative PCR	BA 17, 18, 9, 7	PV, SST	20	20	N
Volk	2012	quantitative PCR and FISH	BA 9	PV, SST, CR	42	42	N
Woo	2008	in situ hybridization	BA 24	CB	20	20	Y
Konradi	2011	qPCR	CA 1, 3, 4	PV, SST	13	11	N
* Data is from the same sample of individuals				Total:	827	800	

The asterisk (*) on sample N’s designates data from the same sample of individuals.

## KEY POINTS

### Question:

What are the alterations in GABAergic interneurons in specific brain regions and cortical layers in ScZ compared to HCs?

### Findings:

This meta-analysis of 46 post-mortem studies showed significant reductions in PV and SST interneurons in the PFC and hippocampus. PV interneuron density was reduced in superficial layers of the PFC, while SST mRNA expression was reduced in layers 2 and 5.

### Meaning:

These findings suggest that specific GABAergic interneuron deficits, particularly PV and SST in the PFC and hippocampus, may contribute the most to the pathophysiology of ScZ.

## Footnotes

a Throughout the results, references to altered ‘density’ pertain specifically to results from IHC and ICC studies, whereas mentions of altered ‘mRNA expression’ relate to results from mRNA-focused studies (from methods such as qPCR, FISH, or in situ hybridization).
