# Cell Density and mRNA Expression of Inhibitory Interneurons in Schizophrenia: A Meta-Analysis

**DOI:** 10.1101/2025.05.23.655812

**Published:** 2025-09-26

**Authors:** Aidan G. Mulvey, Kaitlyn M. Gabhart, Tineke Grent-’t-Jong, Suzana Herculano-Houzel, Peter J. Uhlhaas, André M. Bastos

**Affiliations:** 1 Department of Psychology, Vanderbilt University, Nashville, TN; 2 Department of Child and Adolescent Psychiatry, Charité-Universitätsmedizin, Berlin; 3 Department of Psychology, Yale University, New Haven, CT; 4 Department of Clinical and Counseling Psychology, Columbia University, New York, NY

## Abstract

GABAergic inhibitory interneurons have been implicated in the pathophysiology of schizophrenia. However, there is conflicting evidence regarding the nature and extent of the deficits across brain areas and interneuron subtypes. To address these questions, we conducted a systematic search for immunohistochemistry and mRNA studies that examined parvalbumin, somatostatin, calbindin, and calretinin interneuron density or expression in schizophrenia patients and carried out a meta-analysis using a random-effects model. Data from 25 immunohistochemistry studies (311 control participants, 281 individuals with schizophrenia) revealed that the hippocampus and prefrontal cortex were characterized by GABAergic interneuron deficits; parvalbumin and somatostatin interneuron density was reduced in the hippocampus, while data from 21 mRNA studies (546 control participants, 551 individuals with schizophrenia) indicated reduced parvalbumin and somatostatin expression in the prefrontal cortex and hippocampus. Cortical layer-specific analyses demonstrated that parvalbumin interneuron density and somatostatin mRNA expression were most affected in superficial layers 2 and 3 of prefrontal cortex. We also identified significant reductions in subcortical calretinin interneuron density. Together, these data have important implications for the pathophysiology and computational models of circuit deficits in the disorder.

## Introduction

Schizophrenia (ScZ) is a severe psychiatric disorder characterized by positive symptoms, such as delusions and hallucinations, and negative symptoms including anhedonia and reduced affect (1). In addition, individuals with ScZ have pronounced cognitive deficits involving basic sensory processes as well as dysfunctions in higher cognitive processes (2,3).

Despite significant advances in understanding the pathophysiology of the disorder (4,5), the underlying mechanisms and localization of circuit deficits remain unclear. Thus, different cortical and subcortical areas, including the prefrontal cortex (PFC) (5), hippocampus (6), and thalamus (7) have been implicated in the disorder. In addition, aberrant dopaminergic (8), glutamatergic (9) and γ-aminobutyric acid (GABAergic) (4,10) neurotransmission have been proposed in circuit models of ScZ.

Converging evidence from post-mortem studies suggests that GABAergic interneurons are impaired in ScZ (5,11,12). In fact, altered GABAergic neurotransmission in ScZ is one of the most well replicated findings in the pathophysiology of the disorder (12). GABAergic interneurons are a fundamental aspect of neural circuits due to their role in exerting precise rhythmic inhibition of pyramidal cells thereby leading to the emergence of neural oscillations (13,14). Different subtypes of inhibitory interneurons with distinct morphological and functional characteristics can be distinguished based on their expression of calcium-binding proteins: 1) Parvalbumin (PV) 2) Calbindin (CB) and 3) Calretinin (CR) inhibitory interneurons, as well as based on neuropeptide and neurotransmitters : 1) Somatostatin (SST), 2) vasoactive intestinal peptide, 3) neuropeptide Y, 4) cholecystokinin, and 5) 5-HT3A receptors (12).

Studies using immunohistochemistry (IHC) report deficits in cell density of GABAergic interneurons in ScZ (15–17). In addition, messenger RNA (mRNA) expression reductions of interneuron subtypes have been reported across cortical and subcortical brain regions (5,11,18–20). However, a comprehensive overview of deficits in GABAergic interneurons, the brain regions, and layers involved is not currently available. Previous studies have focused on PV interneurons in the PFC in ScZ (11,21–23), reporting reductions of mRNA expression in layers 2, 3, and 4, but these results are not consistent across studies (17), and evidence suggests these alterations extend to other cortical areas (24,25), as well as to subcortical and hippocampal regions (20,26).

Among the neurophysiological signatures, gamma-band oscillations (30-100 Hz), have been linked to impaired cognition in schizophrenia (27–29). Abnormal brain oscillations have thus been a target for new therapeutics (30). In addition, while biophysical-based computational models offer a promising approach to understanding complex brain dynamics and disruptions, their effectiveness is constrained by the lack of a quantitative and comprehensive neuroanatomical dataset on the inhibitory interneurons most affected in ScZ (31–33).

Accordingly, we conducted a meta-analysis of human post-mortem ScZ studies to examine changes in PV, CB, CR and SST interneurons across cortical, hippocampal, and subcortical regions to provide a novel perspective on this fundamental aspect of the neurobiology of ScZ that could inform models of circuit dysfunctions as well as novel therapeutics. We focused on these PV, CB, CR and SST interneurons subtypes as the link between the other cell types and ScZ is weaker (12,34). Specifically, data from IHC and mRNA studies were analyzed to determine the overall deficits in ScZ, involving reduced neuronal density counts (quantified by IHC studies) and/or altered mRNA expression levels of GABAergic interneurons.

## Methods

### Search strategy and selection criteria

Following PRISMA guidelines (35), ProQuest, PubMed, and Google Scholar were searched with the following search terms: ‘schizophrenia OR ScZ OR SZ AND interneurons’, ‘(interneuron density) AND schizophrenia, ‘immunohistochemistry AND schizophrenia, ‘(layer-specific cell density) AND schizophrenia, ‘(interneuron density) AND (clinical disorders)’, ‘Parvalbumin OR Calbindin OR Calretinin OR Somatostatin AND schizophrenia, ‘(layer-specific interneuron deficit) AND schizophrenia, ‘(mRNA expression) AND schizophrenia, ‘(interneuron mRNA) AND schizophrenia’, and ‘(interneuron expression) AND schizophrenia’.

**Figure 1.**
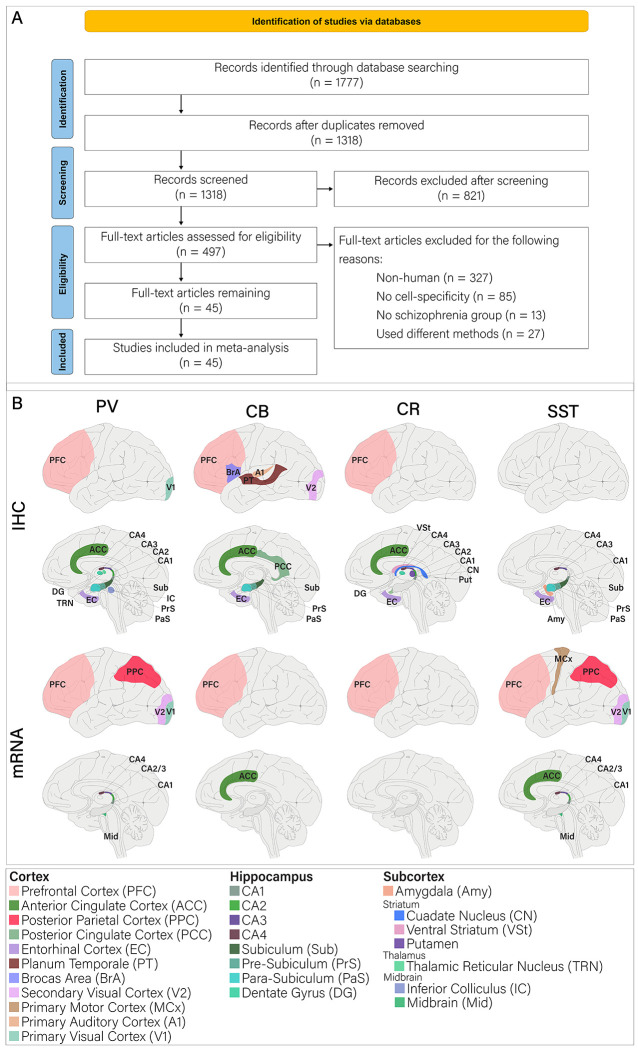
A) PRISMA 2020 flowchart for study screening and inclusion. B) Each brain region that a record analyzed (mRNA and IHC) is shaded in its own designated color. Abbreviations used throughout the present work are given by the Figure 1B legend.

We identified a total of 1,318 studies after duplicate removal and title screening of which 821 studies were excluded for the following reasons (Fig. 1A): 1) 327 studies of non-human samples, 2) 85 studies did not include cell-specific data, 3) 13 studies did not include ScZ groups, 4) 27 studies had different methodologies (e.g., included median instead of mean or did not include individual data points to calculate mean/error). In the meta-analysis of PV studies, papers were excluded if they examined parts of the brain where PV-positive cells are not classified as inhibitory interneurons. We also included data from one published thesis (36) . The final sample included 45 studies that accumulated a total of 857 healthy controls (HCs) and 831 individuals with ScZ. The asterisk (*) in the columns containing sample sizes shows studies that used the same or largely overlapping samples (brains). However, we treated the samples as independent because each sample analyzed different brain regions, cell types, or methods (Table 1).

A quantitative meta-analysis was performed on IHC studies (*n* = 25), where we examined interneuron density for PV (*n* = 18), CB (*n* = 10), CR (*n* = 10), and SST (*n* = 4) studies. Similarly, we examined 20 studies, including one study with both IHC and mRNA data (26) that examined mRNA expression levels for PV (*n* = 16), CB (*n* = 3), CR (*n* = 6), and SST (*n* = 15) studies (Table S1). We included laminar (*n* = 22) and non-laminar data (*n* = 24), with one study including both laminar and non-laminar areas (37). Laminar data were defined as studies that included attainable data (means, SEM) for a specific layer in any cortical region that was collected for a specific cell type. Nonlaminar data included noncortical areas and cortical regions that did not specify a layer where data was collected.

### Data extraction

When papers provided the mean and standard error of the mean (SEM) directly in a table or figure, that data was used. If a paper reported variation via standard deviation (SD) or confidence interval (CI), the SEM was manually calculated using these formulas: $\mathrm{SEM}=SD/\sqrt{n}$, where ‘n’ is the sample size, *SEM* = *CI/zscore*, where z-score is 1.96 for a 95% CI (38). We used WebPlotDigitizer (39) to extract the mean and variation (SEM, SD, or CI) when data was presented in a bar plot or plotted as individual data points (e.g. scatter plot), but not for a table or in text. In 1 study (Wang et. al., 2011), authors were contacted for raw data. Data reporting medians were excluded when a mean could not be calculated. All data was converted to the format of mean +/− SEM to ensure consistency across the meta-analysis. Additionally, when studies used the same subjects, we considered the data points to be independent if the studies analyzed different brain regions, different cell types, or employed different methodologies (e.g., IHC vs mRNA analysis) because each analysis was examining a distinct variable.

The mean and SEM were derived from each layer in each brain region to quantify PV, CB, CR, and SST interneuron densities and mRNA expression. Included studies quantified mRNA expression using grain analysis, optical density analysis, fluorescent in situ hybridization (FISH), or quantitative real-time PCR (qPCR). Importantly, no statistical differences have been identified between FISH and qPCR (40), which we confirmed in our data set with a two-sample t-test for SST interneurons (*p* = 0. 55), but not for PV interneurons (*p* = 0. 04).

### Data synthesis and statistical analysis

We applied a t′-score framework (equation 1), which allows comparison across studies by standardizing the mean differences to their uncertainty values and eliminating any variation in units.

(equation 1), $$t^{\prime }=\frac{a-b}{\sqrt{u{\left(a\right)}^{2}+u{\left(b\right)}^{2}}}$$

in which *a* and *b* are the mean densities/expressions in ScZ and HC, respectively, and *u(a)* and *u(b)* are the SEMs of ScZ and HC, respectively, across individuals within a study. Thus, a negative t’-score indicates a cellular or transcriptional loss in ScZ, and a positive t’-score indicates a cellular or transcriptional gain in ScZ. The t’-score was derived from each cortical layer in each study. If a study combined layers (i.e. L5/6), the resulting t’-score was used for both layers.

24 studies reported whole-area data, instead of laminar-specific results. For such studies, a single t’-score was calculated from the mean and SEM of each area. To best compare effects across areas, a mean t’-score was calculated for each laminar study by combining the laminar means and SEMs into a single mean and SEM for that study for both ScZ and HC, followed by a t’-score. To assess significance, we report the t’-score with the p-value of a one-sample t-test.

**Figure 2.**
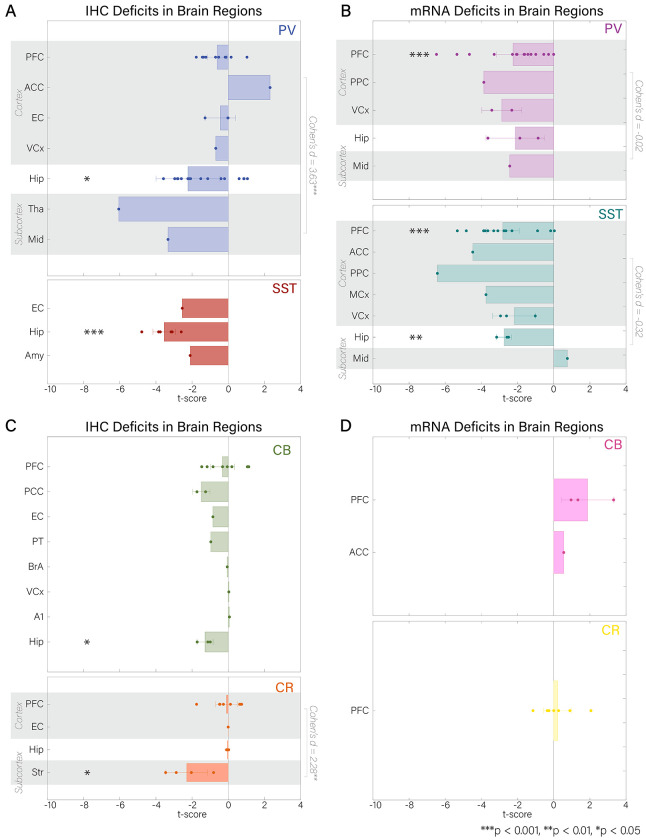
t’-scores for IHC and mRNA methods in all brain regions and cell types that were included in the analysis. Negative t-scores reflect deficits in ScZ. Data points are individual studies. Gray shadings are demarcations of areas included in either cortex or subcortex for effect size analyses (Cohen’s d included). A) Comparison of IHC t-scores for PV and SST interneurons. B) Comparison of mRNA t-scores for PV and SST interneurons. C) Comparison of IHC t-scores for CB and CR interneurons. D) Comparison of mRNA expression t-scores for CB and CR interneurons.

All abbreviations used in the present text are outlined in Figure 1B. PFC was frequently subdivided into Brodmann areas 9, 10, 11, and 46, which are collectively referred to as PFC for the present meta-analysis. Additionally, we will broadly refer to different brain structures as such cortex (PFC, ACC, PPC, PCC, EC, PT, BrA, V2, MCx, A1, V1), hippocampus (CA1-4, Sub, PrS, PaS, and DG), thalamus (TRN), striatum (VSt and Put), midbrain ([Mid] IC and Mid), and subcortex (thalamus, striatum, midbrain, and amygdala [Amy]; Fig. 1B). Thus, if a study quantified multiple structures within a region (i.e. CA1 and CA2), a t′-score was taken for each structure (Fig. 2C) but grouped for regional analysis (Fig. 2A–B; Table S1). To compare the extent of effects, we used a two-sample t-test and Cohen’s effect size analysis on the t′-scores for each region.

### Assessing publication bias and mediating factors

Publication bias was assessed using visual inspection of funnel plots and Egger’s regression testing for all areas and studies for each cell type and method, where *n* > 5 (41). To calculate the standard error of the effect, we used equation 2, where *N* is the number of subjects from the ScZ group (A) control group (B) and *t*’ is the t′-score of the study. The standardized effect size was regressed against the inverse of the standard error (equation 2) and the intercept of this regression (*B*_0_) was then assessed for evidence of publication bias.


(equation 2) $$SE(t^{\prime })=\sqrt{\frac{N_{A}-N_{B}}{N_{A}N_{B}}+\frac{{t^{\prime }}^{2}}{2(N_{A}+N_{B})}}$$

We also performed a one-sample t-test across the data set to determine significant effects of the t’-score (Fig. S3). For biased sets, we removed outlier data points until *p*(*B*_0_)≥0. 05. We conducted a one-sample t′-score testing with a minimum of 5 data points at all times. After removal, we reassessed significance with a one-sample t-test to determine if the bias affected the significance.

We extracted demographic data, where possible, from each study (Table 1). To assess confounds, we used regression models of t′-score and mean age, male-female ratio, post-mortem interval (PMI), and brain pH. For all factors, we took a weighted average of HC and ScZ parameters to get a single value per model input. We report the regression coefficient (*B*_1_) and the Bonnferroni-corrected p-value (*p*(*B*_1_)) of each regression (see Fig. S2A).

## Results

### Alterations of inhibitory interneurons per brain area in IHC and mRNA studies

When considering brain areas and combining sub-area designations and layers, the hippocampus and the PFC were the only areas with significant alterations across studies.^1^ The density of PV (*t*′ =− 2. 23, *df* = 18, *p* < 0. 05), SST (*t*′ =− 3. 54, *df* = 4, *p* < 0. 05), and CB (*t*′ =− 1. 28, *df* = 2, *p* < 0. 05) interneurons were decreased in the hippocampus (Fig. 2A, 5A), as well as mRNA expression for SST (*t*′ =− 2. 75, *df* = 2, *p* < 0. 01; Fig. 2B, 5B). While mRNA expression for PV (*t*′ =− 2. 21, *df* = 16, *p* < 0. 001; Fig. 2B, 5B) and SST (*t*′ =− 2. 70, *df* = 13, *p* < 0. 001) interneurons was reduced in PFC, neuronal density was not reduced for CB (*t*′ =− 0. 32, *df* = 8, *p* > 0. 05), CR (*t*′ =− 0. 10, *df* = 7, *p* > 0. 05), nor PV (*t*′ =− 0. 52, *df* = 9, *p* > 0. 05; Fig. 2A, C, Fig. 5A).

We found reduced density in subcortical structures (Str) for CR interneurons (*t*′ =− 2. 30, *df* = 3, *p* < 0. 05; Fig. 4B, Fig. 5A). Compared to cortical areas (PV: PFC, ACC, EC, V1; CR: PFC, ACC, EC), deficits in subcortical regions (PV: Tha, Mid) were significantly larger for both PV and CR interneuron density (PV: *t*(15) = 5. 03, *p* < 0. 001, *Cohen’s d* = 3. 59; CR: *t*(11) = 4. 08, *p* < 0. 01, *Cohen’s d* = 2. 28; Fig. 2A, C; Fig. 4A; Fig. S1A).

SST mRNA expression studies showed a trend in the opposite direction, with slightly larger effects in cortex compared to subcortex (*t*(19) =− 0. 27, *p* = 0. 79, *Cohen’s d* =− 0. 26; cortex: PFC, PPC, ACC, MCx, V1, V2; subcortex: Mid; Fig. 1B; Fig. 4C; Fig. S1A). mRNA expression of PV interneurons was similar in cortical and subcortical structures (*t*(19) = 0. 04, *p* = 0. 97, *Cohen’s d* = 0. 04; cortex: PFC, PPC, V1, V2; subcortex: Tha, Mid; Fig. 1B; Fig. 4B; Fig. S1A).

We also found significant reductions when taking the mean t′-score across all brain regions for cell density of PV (*t*′ =− 1. 60, *df* = 35, *p* < 0. 01; Fig. 4A), CB (*t*′ =− 0. 60, *df* = 18, *p* < 0. 01; Fig. 4B), and SST (*t*′ =− 3. 24, *df* = 7, *p* < 0. 001; Fig. 4B) and mRNA expression of PV (*t*′ =− 2. 33, *df* = 23, *p* < 0. 001; Fig. 4C) and SST (*t*′ =− 2. 77, *df* = 23, *p* < 0. 001; Fig. 4D), but not for CR density (Fig. 4B), norCB and CR mRNA expression.

### Layer-resolved alterations of inhibitory interneurons in cortex

**Figure 3.**
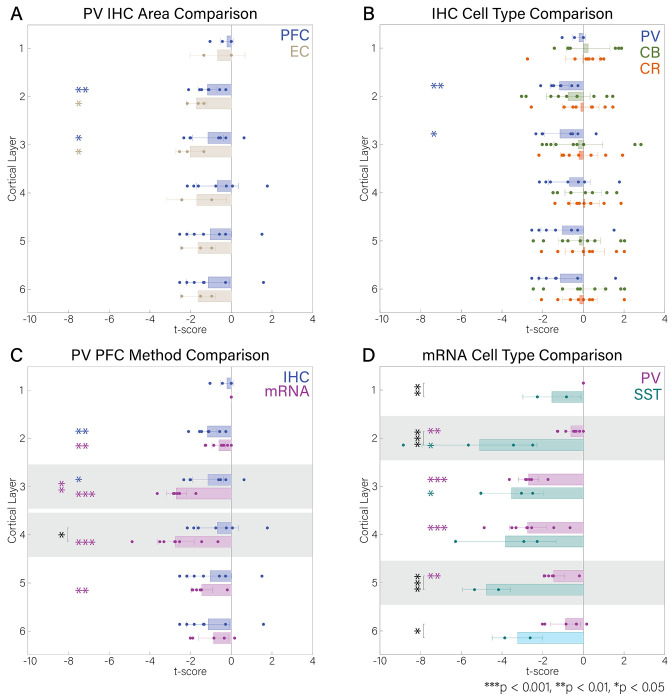
Laminar distributions and comparisons of data points in A) PFC vs. EC in IHC, B) cell type (PV, CB, CR) IHC, C) PV PFC IHC vs. mRNA, and D) PV and SST mRNA. ± 2 SEM is given in error bars. Colored asterisks represent significance at 3 different levels (*** p<0.001, ** p<0.01, *p<0.05) on a one-sample t-test to assess brain areas/cell types that are affected (different between ScZ and HC). Black asterisks with bars represent significance at 3 levels (*** p<0.001, ** p<0.01, *p<0.05) comparing the indicated distributions, with a two-sample t-test.

**Figure 4.**
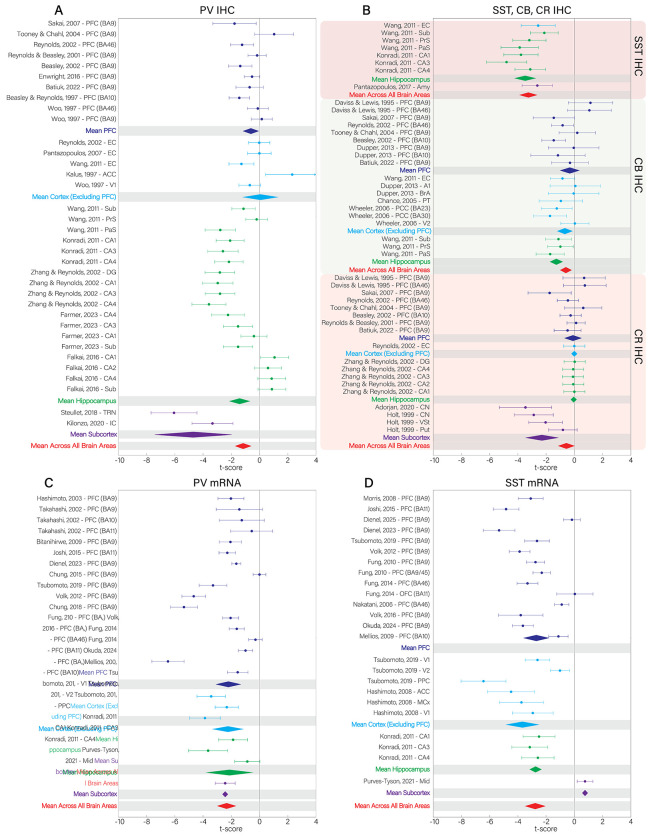
Forest plots of all studies for A) PV IHC, B) SST, CB, and CR IHC, C) PV mRNA, and D) SST mRNA data sets. Dots are the t’-score ± 1 standard error of the effect. Regional mean t’-scores ± 2*SEM are plotted as diamonds. The mean across all brain areas ± 2*SEM is plotted in the red diamond. t’-score of area CA2 from Zhang & Reynolds was removed from subpanel A (t’ = −16.918 ± 4.54), because it is a large outlier.

**Figure 5.**
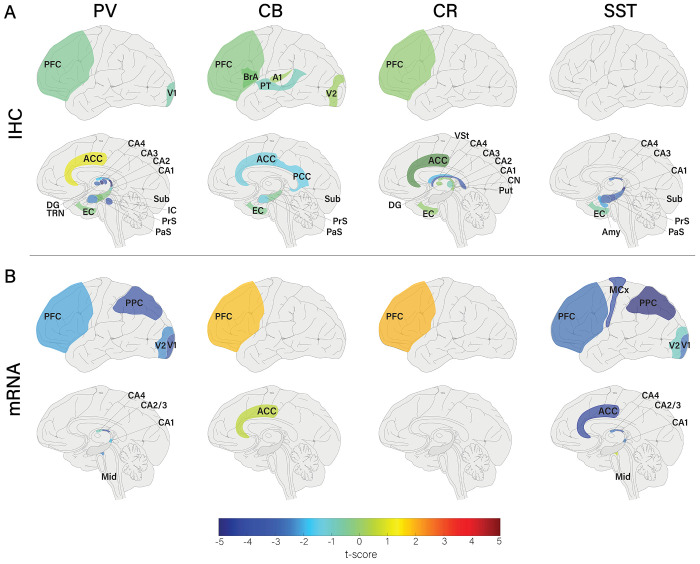
Brain-wide alterations in interneuron density or expression for each cell type, method, and area. A) PV, CB, CR, and SST alterations measured by IHC (cell density). B) PV, CB, CR, and SST alterations in mRNA expression.

Although PFC interneuron density was not affected when all layers were combined (Fig. 2, Fig. 4), PV interneuron density was selectively decreased in L2 (*t*′ =− 1. 17, *df* = 6, *p* < 0. 01; Fig. 3B) and L3 (*t*′ =− 1. 15, *df* = 7, *p* < 0. 05; Fig. 3B). In addition, the EC showed comparable reductions in PV interneuron density in L2 (*t*′ =− 1. 71, *df* = 2, *p* < 0. 05; Fig. 3A) and L3 (*t*′ =− 2. 02, *df* = 2, *p* < 0. 05; Fig. 3A). CB and CR interneurons were not affected in the PFC, both at the whole-area and layer-specific resolution (*p* > 0. 05; Fig. 3B).

Reductions in mRNA expression were more widespread across layers. PV mRNA expression was decreased in PFC in L2-L5, with the strongest effect observed in L4 (L2: *t*′ =− 0. 61, *df* = 7, *p* < 0. 01; L3: *t*′ =− 2. 70, *df* = 5, *p* < 0. 001; L4: *t*′ =− 2. 74, *df* = 7, *p* < 0. 001; L5: *t*′ =− 1. 45, *df* = 5, *p* < 0. 01; Fig. 3C). SST interneuron mRNA expression was reduced in L2 and 3 in PFC, though was slightly less powered than the PV mRNA analysis (L2: *t*′ =− 5. 11, *df* = 3, *p* < 0. 05; L3: *t*′ =− 3. 52, *df* = 2, *p* < 0. 05). Notably, deficits in SST expression were statistically larger than PV expression deficits in L1, 2, 5, and 6 (*L*1: *p* < 0. 01, *L*2: *p* < 0. 001, *L*5: *p* < 0. 001, *L*6: *p* < 0. 05, Fig. 3D).

### Comparison between IHC and mRNA Expression Studies

mRNA expression of PV interneurons was more affected than PV interneuron density in PFC (*t*(25) = 2. 64, *p* < 0. 05, *Cohen’s d* = 1. 02; Fig. S1B), particularly in L3 and L4 (*p* < 0. 01 and *p* < 0. 05, respectively; Fig. 3C, Fig. S1C). However, deficits in mRNA expression and neuronal density were comparable in hippocampal PV interneurons (*t*(20) =− 0. 04, *p* = 0. 97, *Cohen’s d* =− 0. 03; Fig. S1B) and SST interneurons (*t*(7) =− 1. 67, *p* = 0. 13, *Cohen’s d* =− 1. 05; Fig. S1B).

### Publication bias and mediating factors

Our analysis of publication bias using funnel plots and Egger’s test determined that data sets for PV (*B*_0_ =− 6.42, *df* = 35, *p* < 0. 001), CR (*B*_0_ =− 3. 66, *df* = 17, *p* < 0. 05), and SST (*B*_0_ =− 11. 12, *df* = 7, *p* < 0. 01) interneuron density exhibited evidence of publication bias (Fig. S3A, B, C). We also found evidence of publication bias in the mRNA expression data sets for PV (*B*_0_ =− 5.13, *df* = 23, *p* < 0. 01) and SST (*B*_0_ =− 7. 69, *df* = 23, *p* < 0. 01) interneurons (Fig. S3E, G). CB interneuron density and CR mRNA expression data sets remained unbiased. There were not enough data points (fewer than 5 data points) to perform this analysis on the CB mRNA expression data set. To correct for this identified bias, we removed outlying data points for each biased data set (circled dots in Fig. S3). When we reassessed significance, no cell type for either density or mRNA expression data sets lost or gained significance.

We also tested for whether the reported effects could have been affected by age, sex, brain pH, and post-mortem interval (PMI). After Bonferroni correction (*n* = 28 regressions), we found no significant correlations between these factors and PV IHC, SST IHC, and CR mRNA (regressions, all *p* > 0. 05, Bonferroni corrected for 28 regressions, Fig. S4). The regressions of mediating factors revealed significant relationships between CR interneuron density t’-score and male-female ratio (*B*_1_ =− 2. 54, *p* < 0. 0018) (Fig. S4B, C, E, F). There was insufficient data for all regressors of CB mRNA expression, as well as for brain pH and SST interneuron density (fewer than 5 studies).

Finally, it is important to consider medication status. Of the 45 studies in our meta-analysis, 9 reported data on Chlorpromazine (CPZ) equivalent doses. All studies overlapped in their reported ranges of CPZ-doses.

## Discussion

These data provide the first comprehensive meta-analysis of changes in GABAergic interneurons from post-mortem data in ScZ. Our results show that alterations in GABAergic interneurons were consistently observed in the hippocampus and PFC. Among the subtypes of GABAergic interneurons, PV and SST interneurons were particularly affected in ScZ. While CB cell density was overall reduced, CR was mostly intact. Layer-specific analyses of PFC revealed PV deficits were most pronounced in superficial L2/3, while SST interneurons exhibited the strongest deficits in superficial L2.

Current theories have implicated different cortical and subcortical brain regions in ScZ with deficits in GABAergic interneurons reported across frontal (17) and sensory areas, (36,42–44) hippocampus, (26,45–47) as well as the basal ganglia (48) and the limbic system (10). The current findings highlight that GABAergic interneurons in the hippocampus and PFC are characterized by the largest and most consistent alterations. Circuit deficits in the PFC have been implicated in a range of cognitive deficits in ScZ, in particular working memory (49) and executive functions (50,51). Impaired hippocampal functioning has been proposed as an early signature of psychosis (52–54) and could also be related to reduced functionality of the PFC (55), and impaired hippocampal–prefrontal connectivity (56).

PV and SST subtypes were particularly affected/altered in ScZ, which is consistent with genomics and transcriptomics work that have identified risk genes associated with these cell types in ScZ (57,58). In addition, there is consistent evidence of PV deficits in animal models of ScZ in PFC and hippocampus (59). In a mouse model of ScZ, optogenetic stimulation of PV interneurons in the PFC and hippocampus rescued schizophrenia-like symptoms (60).

PV interneurons are critically involved in the generation of gamma-band (30-100 Hz) oscillations (61–63), which are impaired in ScZ (for a review, see Uhlhaas & Singer, 2010) (28). SST interneurons have also been associated with the generation of gamma-band oscillations (61,63,64) but also with slower frequencies, including theta band (4-6 Hz) (65,66) and beta band (15-30 Hz) (67) oscillations.

Moreover, we found significant reductions in PV and SST mRNA expression across cortical layers in PFC. PV mRNA expression was most reduced in L3/4, where this cell type is highly enriched (68). The mRNA deficits of SST interneurons spanned L2/3 and L5. These layer-specific deficits, paired with the deficits uncovered at the whole-area resolution, have implications for computational models of ScZ.

One class of influential models is the predictive coding approach (8,69,70). According to these models, prediction errors are computed by L2/3 pyramidal neurons (71), which are under the inhibitory control of local interneurons in these layers. Deficits in prediction errors in ScZ could underlie aberrant sensory inferences, leading to delusions and hallucinations (70). In addition, gamma-band oscillations in superficial layers (L2/3) support feedforward prediction error propagation (72–76). Together these data suggest that impaired GABAergic interneurons in ScZ could lead to impaired feedforward processing (and reduced prediction error) as well as reduced which could contribute towards hallucinations and delusions and possibly also cognitive deficits.

Our results from IHC and mRNA studies provided converging but also distinct perspectives on GABAergic interneuron deficits in ScZ. Interneuron density in subcortical structures was more reduced than in cortical regions for PV and CR interneurons. We found the opposite trend in mRNA expression of SST interneurons, with PV mRNA expression being equally affected in both cortical and subcortical regions. It should also be noted that ScZ may be characterized primarily by loss of mRNA without loss of neurons in many structures (11). Therefore, it is possible that subcortical PV and CR interneurons are more vulnerable to aberrant developmental processes in ScZ compared to their cortical counterparts. Future studies could examine the distinctions between these cortical and subcortical interneurons cells in greater detail.

## Limitations

Several limitations warrant consideration when interpreting this meta-analysis. Firstly, the included studies exhibited unequal sampling across different brain regions, cell types, and cortical layers. This could be addressed in future studies with a broader and more equal sampling across the brain. Second, these results cannot definitively determine whether the observed PV and SST interneuron deficits represent a primary pathology in ScZ or arise secondary to other factors, such as impairments in excitatory neurotransmission (for a review, see Dienel et al., 2022) (77). Moreover, some aspects of the data were underpowered; however, we continued with these analyses to provide a comprehensive view of the data.

## Summary

This meta-analysis with over 1,688 participants, 25 brain regions, and 4 inhibitory cell types provides the first comprehensive overview of GABAergic interneuron deficits in ScZ from post-mortem data, with implications for the pathophysiology of the disorder. The convergence on PV and SST interneuron deficits in PFC and hippocampal circuits is important for current computational and circuit models (66,78). These findings also suggest that the GABAergic etiology of ScZ is highly specific to brain regions and cortical layers, which highlights the need for the present comprehensive study. Future investigations should also explore the functional consequences of layer-specific deficits and their contribution to cognitive impairments in ScZ, as well as the relationship to other aspects of circuit deficits, such as impaired excitatory neurotransmission (79,80). Additionally, these results could guide the development of targeted interventions aimed at restoring the function of PV and SST interneurons through pharmacological (81) or neuromodulatory (30) approaches.

## Supplementary Material

Supplement 1

Supplement 2

## Figures and Tables

**Table 1.** All included papers. First author, publication year, method(s), brain area(s), cell type(s), number of subjects (N) for both healthy control (HC) and schizophrenia (SCZ) groups, if the study did (Y) or did not (N) examine cortical layers.

Author	Year	Method	Area	Cell Type	N HC	N SCZ	Laminar
**Density**							
Sakai	2007	ICC	BA 9	PV, CB, CR	5	7	Y
Tooney & Chahl	2004	IHC	BA 9	PV, CB, CR	6	6	Y
Reynolds	2002	ICC	BA 46	PV, CB, CR	15	15	Y
			BA 28	PV, CR			
Beasley and Reynolds	1997	IHC	BA 10	PV	22*	18*	Y
Beasley	2002	ICC	BA 9	PV, CB, CR	15	15	Y
Reynolds and Beasley	2001	IHC	BA 9	PV, CR	22*	18*	Y
Enwright	2016	IHC	BA 9	PV	28	28	Y (L3)
Batiuk	2022	IHC	BA 9	PV, CR	10	10	Y
Pantazopoulos	2007	ICC	BA 28	PV	16	10	N
Woo	1997	ICC	BA 17, 9, 46	PV	15	15	N
Kalus	1997	IHC	BA 24	PV	5	5	Y
Daviss & Lewis	1995	IHC	BA 9, 46	CB, CR	5	5	Y
Dupper	2013	IHC	BA 9, 46, 41, 44	CB	5	3	Y
Chance	2005	IHC	BA 22	CB	12	12	Y (L3)
Wang	2011	ICC	BA 28	PV, SST, CB	17	12	Y
			Sub, PrS, PaS	PV, SST, CB			N
Wheeler	2006	IHC	BA 23, 30, 18	CB	9	9	N
Steullet	2018	IHC	TRN	PV	20	15	N
Konradi	2011	ICC	CA 1, 2/3, 4	SST, PV	20	13	N
Zhang and Reynolds	2002	IHC	DG, CA1-4	PV, CR	15	15	N
Farmer	2023	IHC	CA1-4, Sub	PV	11	10	N
Falkai	2016	ICC	CA1, 2/3, 4, Sub	PV	10	10	N
Kilonzo	2020	IHC	IC	PV	20	20	N
Adorjan	2020	IHC	CN	CR	6	6	N
Holt	1999	IHC	CN, VStr, Put	CR	9	10	N
Pantazopoulos	2017	ICC	Amygdala	SST	15	12	N
**mRNA**							
Dienel	2025	fluorescence in situ hybridization (FISH)	BA 9	SST	46	46	Y
Bitanihirwe	2009	FISH	BA 9	PV	20	20	Y
Dienel	2023	in situ hybridization (ISH)	BA 9	PV, SST	30	30	Y
Joshi	2015	ISH, quantitative PCR (qPCR)	BA 11	PV, SST	38	38	Y
Morris	2008	ISH	BA 9	SST	23	23	Y
Takahashi	2002	ISH	BA 9, 10, 11	PV	5	6	Y
Hashimoto	2003	Optical density (OD)	BA 9	PV, CR	15	15	Y
Fung	2010	qPCR	BA 46	PV, SST, CB, CR	37	37	N
Nakatani	2006	qPCR	BA 46	SST	7	7	N
Volk	2016	qPCR	BA 9	PV, SST	39	39	N
Purves-Tyson	2021	qPCR	Midbrain	PV, SST	28	28	N
Fung	2014	ISH	BA 46, 11	PV, CB, CR, SST	34	35	N
Chung	2018	qPCR	BA 9	PV	40	40	N
Tsubomoto	2019	qPCR	BA 17, 18, 9, 7	PV, SST	20	20	N
Volk	2012	qPCR	BA 9	PV, SST, CR	42	42	N
Woo	2008	qPCR, FISH	BA 24	CB	20	20	N
Konradi	2011	qPCR	CA 1, 2/3, 4	PV, SST	13	11	Y
Hashimoto	2008	qPCR	BA 9, 17, 24, 4	SST	12	12	N
Okuda	2024	qPCR	BA 9	PV, SST, CR	40	40	N
Chung	2015	qPCR	BA 9	PV, CR	39	39	Y
Mellios	2009	qPCR	BA 10	PV, SST	20	20	N
				Total:	857	831	

* Data is from the same sample of individuals

## Footnotes

1 Throughout the results, references to altered ‘density’ pertain specifically to results from IHC and ICC studies, whereas mentions of altered ‘mRNA expression’ relate to results from mRNA-focused studies (from methods such as qPCR, FISH, or in situ hybridization).
