# Supplementary material for: Cell Density and mRNA Expression of Inhibitory Interneurons in Schizophrenia: A Meta-Analysis
Source: bioRxiv. 2025 Sep 26:2025.05.23.655812. Originally published 2025 May 27. Preprint. [Version 2] doi: 10.1101/2025.05.23.655812 (PMC12154732; doi:10.1101/2025.05.23.655812)
Supplement: Supplement 1 [file media-1.pdf]

Supplementary Information

Cell Density and mRNA Expression of Inhibitory Interneurons in Schizophrenia: A Meta-Analysis

Aidan G. Mulvey, B.A.[1, 3†], Kaitlyn M. Gabhart, B.S.[1, 4†], Tineke Grent-'t-Jong, Ph.D.[2], Suzana Herculano-Houzel, Ph.D.[1], Peter J. Uhlhaas, Ph.D.[2‡], André M. Bastos, Ph.D.[1‡✉]

[1]Department of Psychology, Vanderbilt University, Nashville, TN
[2]Department of Child and Adolescent Psychiatry, Charité-Universitätsmedizin, Berlin
[3]Department of Psychology, Yale University, New Haven, CT
[4]Teachers College, Columbia University, New York, NY
[†]These authors contributed equally to this work
[‡]Co-senior authors
[✉]Corresponding author

Corresponding author is Dr. André M. Bastos, Vanderbilt University
301 Wilson Hall, 111 21st Ave S, Nashville TN 37203
Andre.bastos@vanderbilt.edu, 617-710-3468

This file includes:
Supplementary Table 1 Caption
Supplementary Figs. 1-3
References

Supplementary Table 1 Caption

Table S1: Table of all included papers. Papers measuring interneuron density with immunohistochemistry: (1–25). Papers using mRNA expression to measure interneuron mRNA expression include: (18,26–45). The table also includes a link to the paper, first author, publication year, and method. Sub-method indicate the specific method (e.g. neurons/mm^3, mRNA levels [nCi/g]). We also collected significant qualitative findings from the included papers as direction of results (↑-increase, ↓- decrease, ↔-unchaged). We also included the medication status, sex, mean age ± SD in years, brain pH, post-mortem interval (PMI), and RNA integrity number (RIN) for both healthy control (HC) and schizophrenia (SZ) groups. The tissue obtained column indicates the tissue bank(s) from which each study collected samples (if reported). We also included from which figure or table we extracted the data, and the method of data extraction (e.g. direct from a table or WebPlotDigitizer manual extraction). Finally, we included the resulting t'-score and standard error of the t'-score from each study for data availability purposes.

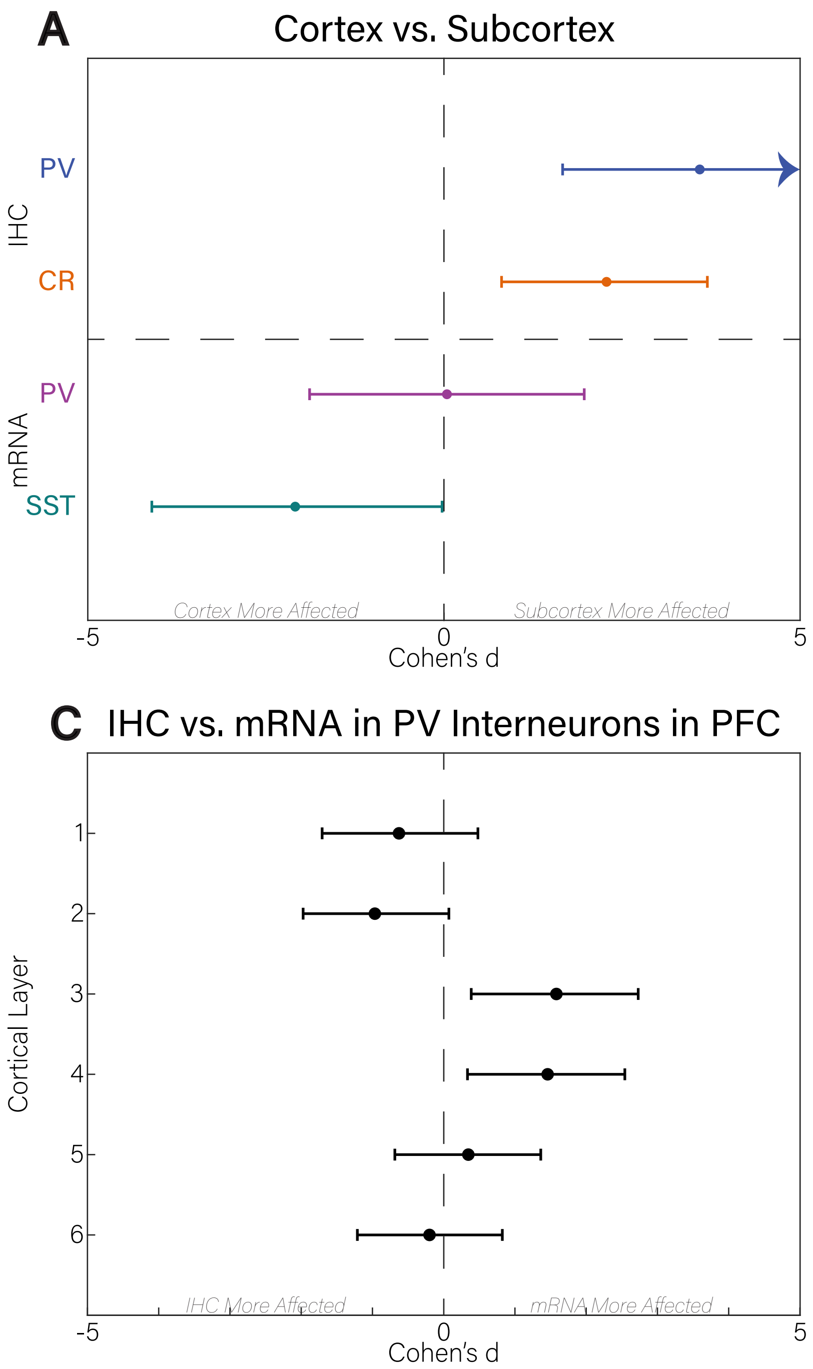

Figure S1. Effect size analysis using Cohen's d on A) cortex versus subcortex in IHC and mRNA for PV, CR, and SST interneurons, B) IHC versus mRNA across brain regions for PV and SST interneurons, and C) IHC versus mRNA for each cortical layer in PV interneurons in the PFC.

A

		Age			Male-Female Ratio			Brain pH			PMI		
		B_1	n	p	B_1	n	p	B_1	n	p	B_1	n	p
IHC	PV	0.01	26	p>0.0018	0.61	30	p>0.0018	7.20	11	p>0.0018	-0.12	26	p>0.0018
IHC	CB	0.01	18	p>0.0018	-0.05	15	p>0.0018	2.20	4	p>0.0018	-0.10	12	p>0.0018
IHC	CR	-0.09	12	p>0.0018	**-2.55**	**15**	**p<0.0018**	-21.5	9	p>0.0018	0.01	12	p>0.0018
IHC	SST	0.15	8	p>0.0018	-3.04	8	p>0.0018	-0.29	1	-	-0.45	8	p>0.0018
mRNA	PV	0.00	20	p>0.0018	0.25	24	p>0.0018	-7.98	23	p>0.0018	-0.00	20	p>0.0018
mRNA	CR	-0.21	7	p>0.0018	1.44	7	p>0.0018	3.28	7	p>0.0018	-0.06	7	p>0.0018
mRNA	SST	0.04	20	p>0.0018	0.07	20	p>0.0018	-10.9	18	p>0.0018	0.08	20	p>0.0018

B

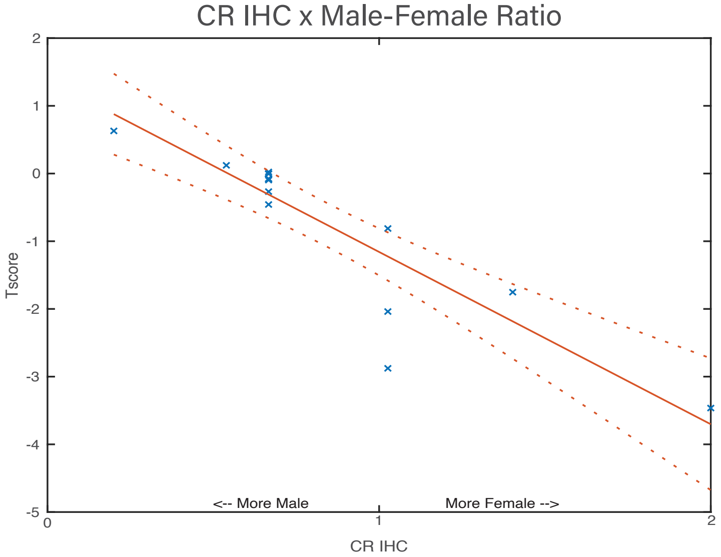

Figure S2. (A) Table of regressor values, number of observations (data points), and significance determined by $p < 0.0018$ (Bonferroni correction = 0.05 / 28) is in bold. Studies with insufficient regressor data (n < 5 observations) were not included. (B) Correlation analysis with a linear regression fit (red lines) and 95% confidence intervals (red dotted lines) show the relationship between mediating factors sex-ratio and CR IHC.

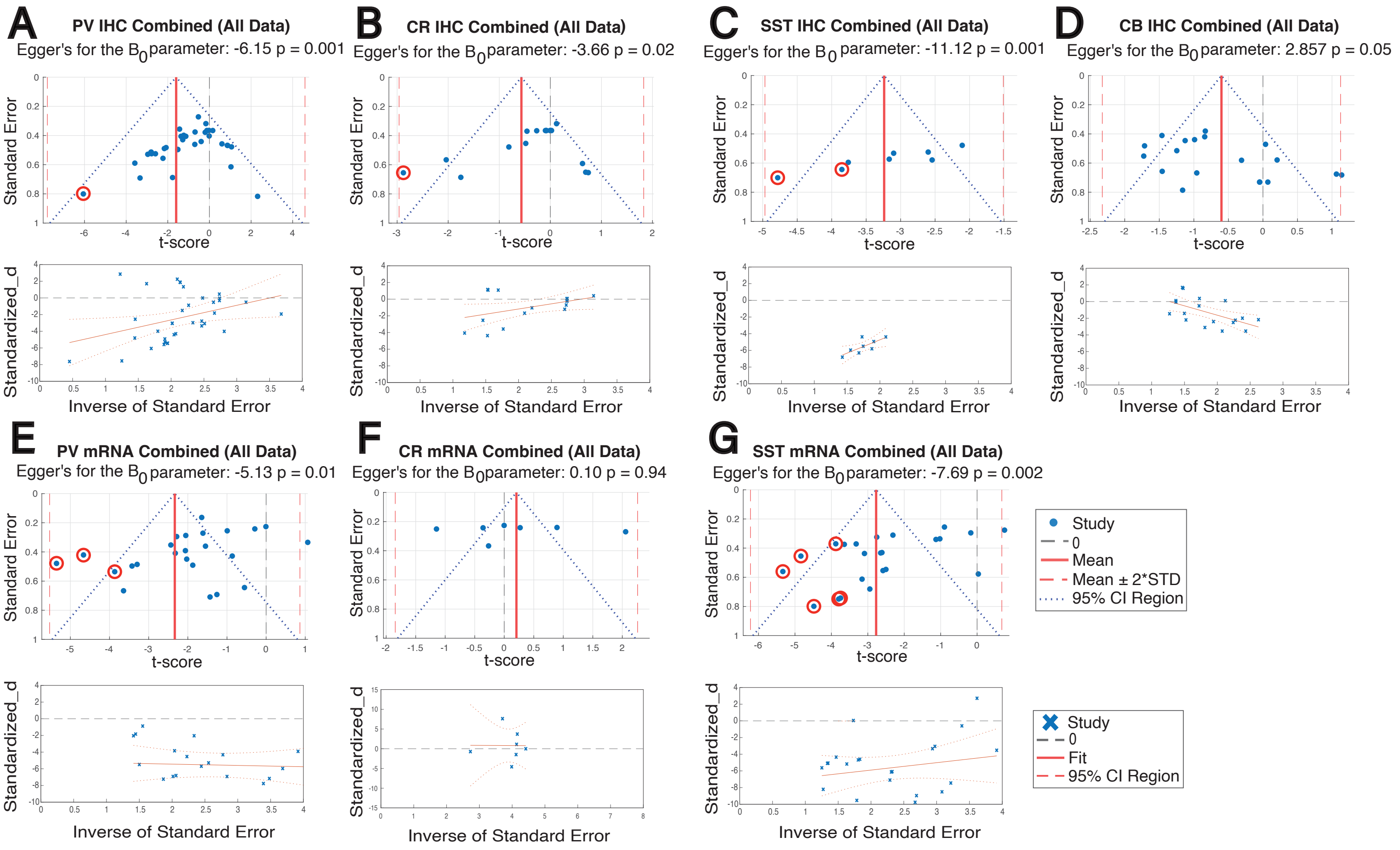

H

		Prior to Bias Correction: Egger's Test B_0	df	p_{B_0}	t′	$p_{t\text{-test}}$	studies removed	After Bias Correction: Egger's Test B_0	df	p_{B_0}	t′	$p_{t\text{-test}}$
PV	IHC	-6.42	35	p<0.001	-1.60	p<0.01	2	-3.98	33	p>0.05	-1.02	p<0.001
	mRNA	-5.13	23	p<0.01	-2.33	p<0.001	4	-2.69	19	p>0.05	-1.78	p<0.001
CB	IHC	2.86	18	p>0.05	-0.60	p<0.01	-	-	-	-	-	-
	mRNA	12.14	3	p>0.05	1.54	p>0.05	-	-	-	-	-	-
CR	IHC	-3.66	17	p<0.05	-0.57	p>0.05	1	-2.67	16	p>0.05	-0.40	p>0.05
	mRNA	1.00	6	p>0.05	0.207	p>0.05	-	-	-	-	-	-
SST	IHC	-11.12	7	p<0.01	-3.24	p<0.001	2	-10.17	5	p>0.05	-2.88	p<0.001
	mRNA	-7.69	23	p>0.01	-2.77	p<0.001	7	-6.63	17	p=0.05	-2.00	p<0.001

Figure S3. Funnel plots (A-G) display the assessment of potential publication bias using Egger's test. For our analysis, we used a minimum of 5 studies. The x-axis represents the effect size (t-score), while the y-axis indicates standard error (SE). Black circles within the funnel plots highlight outlier studies that were removed to de-bias the distribution. Red circles indicate removed studies to correct for publication bias. The following studies were removed, but are not visible on the plot: PV IHC (A), one study (t-score = -16.9184, Author(s) = Zhang and Reynolds, Area(s) = CA2); CR IHC (B), one study (t-score = -3.4641, Author(s) = Adorjan, Area(s) = CN); PV mRNA (E) one study (t-score = -6.4864, Author(s) = Okuda, 2024, Area(s) = PFC); SST mRNA (G), one study (t-score = -6.4371, Author(s) = Tsubomoto, Area(s) = PPC). The subplots below the Funnel plots display the regression analysis corresponding to the Egger's test. The x-axis represents precision (1/SE), and the y-axis represents the standardized effect size. Also displayed are the linear regression fit (red lines) and 95% confidence intervals (red dotted lines). (H) Table of results from Egger's tests, mean t'-score of each data set, and one-sample t-test of the data sets before and after bias correction. The number of studies removed to correct for bias is also included.

Supplementary references

1. Sakai T, Oshima A, Nozaki Y, Ida I, Haga C, Akiyama H, et al. Changes in density of calcium-binding-protein-immunoreactive GABAergic neurons in prefrontal cortex in schizophrenia and bipolar disorder. Neuropathology. 2007 Dec 7;28(2):143–50.

2. Tooney PA, Chahl LA. Neurons expressing calcium-binding proteins in the prefrontal cortex in schizophrenia. Progress in Neuro-Psychopharmacology and Biological Psychiatry. 2004 Mar 1;28(2):273–8.

3. Reynolds GP, Beasley CL, Zhang ZJ. Understanding the neurotransmitter pathology of schizophrenia: selective deficits of subtypes of cortical GABAergic neurons. J Neural Transm. 2002 May 1;109(5):881–9.

4. Reynolds GP, Beasley CL. GABAergic neuronal subtypes in the human frontal cortex — development and deficits in schizophrenia. Journal of Chemical Neuroanatomy. 2001 July 1;22(1):95–100.

5. Beasley CL, Zhang ZJ, Patten I, Reynolds GP. Selective deficits in prefrontal cortical GABAergic neurons in schizophrenia defined by the presence of calcium-binding proteins. Biological Psychiatry. 2002 Oct 1;52(7):708–15.

6. Enwright JF, Sanapala S, Foglio A, Berry R, Fish KN, Lewis DA. Reduced Labeling of Parvalbumin Neurons and Perineuronal Nets in the Dorsolateral Prefrontal Cortex of Subjects with Schizophrenia. Neuropsychopharmacol. 2016 Aug;41(9):2206–14.

7. Batiuk MY, Tyler T, Dragicevic K, Mei S, Rydbirk R, Petukhov V, et al. Upper cortical layer–driven network impairment in schizophrenia. Sci Adv. 2022 Oct 14;8(41):eabn8367.

8. Pantazopoulos H, Lange N, Baldessarini RJ, Berretta S. Parvalbumin Neurons in the Entorhinal Cortex of Subjects Diagnosed With Bipolar Disorder or Schizophrenia. Biological Psychiatry. 2007 Mar 1;61(5):640–52.

9. Tsung-Ung Woo, Jed L. Miller, David A. Lewis. Schizophrenia and the parvalbumin-containing class of cortical local circuit neurons. AJP. 1997 July 1;154(7):1013–5.

10. Kalus P, Bondzio J, Federspiel A, Müller TJ, Zuschratter W. Cell-type specific alterations of cortical interneurons in schizophrenic patients. NeuroReport. 2002 Apr 16;13(5):713.

11. Daviss SR, Lewis DA. Local circuit neurons of the prefrontal cortex in schizophrenia: selective increase in the density of calbindin-immunoreactive neurons. Psychiatry Res. 1995 Nov 29;59(1–2):81–96.

12. Dupper AC. Altered cortical calbindin-immunoreactive interneuron populations associated with schizophrenia. Kent State University; 2013.

13. Chance SA, Walker M, Crow TJ. Reduced density of calbindin-immunoreactive interneurons in the planum temporale in schizophrenia. Brain Research. 2005 June 7;1046(1):32–7.

14. Beasley CL, Reynolds GP. Parvalbumin-immunoreactive neurons are reduced in the prefrontal cortex of schizophrenics. Schizophrenia Research. 1997 Apr 11;24(3):349–55.

15. Wang AY, Lohmann KM, Yang CK, Zimmerman EI, Pantazopoulos H, Herring N, et al. Bipolar disorder type 1 and schizophrenia are accompanied by decreased density of parvalbumin- and somatostatin-positive interneurons in the parahippocampal region. Acta Neuropathol. 2011 Nov;122(5):615–26.

16. Wheeler DG, Dixon G, Harper CG. No differences in calcium-binding protein immunoreactivity in the posterior cingulate and visual cortex: Schizophrenia and controls. Progress in Neuro-Psychopharmacology and Biological Psychiatry. 2006 June 1;30(4):630–9.

17. Steullet P, Cabungcal JH, Bukhari SA, Ardelt MI, Pantazopoulos H, Hamati F, et al. The thalamic reticular nucleus in schizophrenia and bipolar disorder: role of parvalbumin-expressing neuron networks and oxidative stress. Mol Psychiatry. 2018 Oct;23(10):2057–65.

18. Konradi C, Yang CK, Zimmerman EI, Lohmann KM, Gresch P, Pantazopoulos H, et al. Hippocampal interneurons are abnormal in schizophrenia. Schizophrenia Research. 2011 Sept 1;131(1):165–73.

19. Zhang ZJ, Reynolds GP. A selective decrease in the relative density of parvalbumin-immunoreactive neurons in the hippocampus in schizophrenia. Schizophrenia Research. 2002 May;55(1–2):1–10.

20. Farmer CB, Roach EL, Bice LR, Falgout ME, Mata KG, Roche JK, et al. Excitatory and inhibitory imbalances in the trisynaptic pathway in the hippocampus in schizophrenia: a postmortem ultrastructural study. J Neural Transm (Vienna). 2023 July;130(7):949–65.

21. Falkai P, Steiner J, Malchow B, Shariati J, Knaus A, Bernstein HG, et al. Oligodendrocyte and Interneuron Density in Hippocampal Subfields in Schizophrenia and Association of Oligodendrocyte Number with Cognitive Deficits. Front Cell Neurosci [Internet]. 2016 Mar 30 [cited 2024 Mar 28];10. Available from: https://www.frontiersin.org/articles/10.3389/fncel.2016.00078

22. Kilonzo VW, Sweet RA, Glausier JR, Pitts MW. Deficits in Glutamic Acid Decarboxylase 67 Immunoreactivity, Parvalbumin Interneurons, and Perineuronal Nets in the Inferior Colliculus of Subjects With Schizophrenia. Schizophr Bull. 2020 July 18;46(5):1053–9.

23. Adorjan I, Sun B, Feher V, Tyler T, Veres D, Chance SA, et al. Evidence for Decreased Density of Calretinin-Immunopositive Neurons in the Caudate Nucleus in Patients With Schizophrenia. Front Neuroanat [Internet]. 2020 Nov 13 [cited 2024 Sept 3];14. Available from: https://www.frontiersin.org/journals/neuroanatomy/articles/10.3389/fnana.2020.581685/full

24. Holt DJ, Herman MM, Hyde TM, Kleinman JE, Sinton CM, German DC, et al. Evidence for a deficit in cholinergic interneurons in the striatum in schizophrenia. Neuroscience. 1999 Sept 1;94(1):21–31.

25. Pantazopoulos H, Wiseman JT, Markota M, Ehrenfeld L, Berretta S. Decreased Numbers of Somatostatin-Expressing Neurons in the Amygdala of Subjects With Bipolar Disorder or Schizophrenia: Relationship to Circadian Rhythms. Biological Psychiatry. 2017 Mar 15;81(6):536–47.

26. Dienel SJ, Wade KL, Fish KN, Lewis DA. Alterations in Prefrontal Cortical Somatostatin Neurons in Schizophrenia: Evidence for Weaker Inhibition of Pyramidal Neuron Dendrites. Biological Psychiatry [Internet]. 2025 Jan 21 [cited 2025 Mar 12]; Available from: https://www.sciencedirect.com/science/article/pii/S0006322325000526

27. Bitanihirwe B, Lim M, Kelley J, Kaneko T, Woo T. Glutamatergic deficits and parvalbumin-containing inhibitory neurons in the prefrontal cortex in schizophrenia. BMC Psychiatry. 2009 Nov 16;9(1):71.

28. Dienel SJ, Fish KN, Lewis DA. The Nature of Prefrontal Cortical GABA Neuron Alterations in Schizophrenia: Markedly Lower Somatostatin and Parvalbumin Gene Expression Without Missing Neurons. AJP. 2023 July 1;180(7):495–507.

29. Joshi D, Catts VS, Olaya JC, Shannon Weickert C. Relationship between somatostatin and death receptor expression in the orbital frontal cortex in schizophrenia: a postmortem brain mRNA study. npj Schizophr. 2015 Mar 4;1(1):1–9.

30. Morris HM, Hashimoto T, Lewis DA. Alterations in Somatostatin mRNA Expression in the Dorsolateral Prefrontal Cortex of Subjects with Schizophrenia or Schizoaffective Disorder. Cerebral Cortex. 2008 July 1;18(7):1575–87.

31. Takahashi T, Emson PC, Arai H. Region-specific and Age-related Decrease of Parvalbumin Gene Expression in the Prefrontal Cortex of Elderly Patients with Schizophrenia. Psychogeriatrics. 2002;2(1):26–34.

32. Hashimoto T, Volk DW, Eggan SM, Mirnics K, Pierri JN, Sun Z, et al. Gene Expression Deficits in a Subclass of GABA Neurons in the Prefrontal Cortex of Subjects with Schizophrenia. J Neurosci. 2003 July 16;23(15):6315–26.

33. Fung SJ, Webster MJ, Sivagnanasundaram S, Duncan C, Elashoff M, Weickert CS. Expression of interneuron markers in the dorsolateral prefrontal cortex of the developing human and in schizophrenia. Am J Psychiatry. 2010 Dec;167(12):1479–88.

34. Nakatani N, Hattori E, Ohnishi T, Dean B, Iwayama Y, Matsumoto I, et al. Genome-wide expression analysis detects eight genes with robust alterations specific to bipolar I disorder: relevance to neuronal network perturbation. Hum Mol Genet. 2006 June 15;15(12):1949–62.

35. Volk DW, Sampson AR, Zhang Y, Edelson JR, Lewis DA. Cortical GABA markers identify a molecular subtype of psychotic and bipolar disorders. Psychological Medicine. 2016 Sept;46(12):2501–12.

36. Purves-Tyson TD, Brown AM, Weissleder C, Rothmond DA, Shannon Weickert C. Reductions in midbrain GABAergic and dopamine neuron markers are linked in schizophrenia. Molecular Brain. 2021 June 26;14(1):96.

37. Fung SJ, Fillman SG, Webster MJ, Shannon Weickert C. Schizophrenia and bipolar disorder show both common and distinct changes in cortical interneuron markers. Schizophrenia Research. 2014 May 1;155(1):26–30.

38. Chung DW, Chung Y, Bazmi HH, Lewis DA. Altered ErbB4 splicing and cortical parvalbumin interneuron dysfunction in schizophrenia and mood disorders. Neuropsychopharmacol. 2018 Nov;43(12):2478–86.

39. Tsubomoto M, Kawabata R, Zhu X, Minabe Y, Chen K, Lewis DA, et al. Expression of Transcripts Selective for GABA Neuron Subpopulations across the Cortical Visuospatial Working Memory Network in the Healthy State and Schizophrenia. Cereb Cortex. 2019 July 22;29(8):3540–50.

40. Volk DW, Matsubara T, Li S, Sengupta EJ, Georgiev D, Minabe Y, et al. Deficits in Transcriptional Regulators of Cortical Parvalbumin Neurons in Schizophrenia. AJP. 2012 Oct;169(10):1082–91.

41. Woo TUW, Shrestha K, Lamb D, Minns MM, Benes FM. *N*-Methyl-D-Aspartate Receptor and Calbindin-Containing Neurons in the Anterior Cingulate Cortex in Schizophrenia and Bipolar Disorder. Biological Psychiatry. 2008 Nov 1;64(9):803–9.

42. Hashimoto T, Bazmi HH, Mirnics K, Wu Q, Sampson AR, Lewis DA. Conserved Regional Patterns of GABA-Related Transcript Expression in the Neocortex of Subjects With Schizophrenia. AJP. 2008 Apr;165(4):479–89.

43. Okuda T, Kimoto S, Kawabata R, Bian Y, Tsubomoto M, Okamura K, et al. Alterations in inhibitory neuron subtype-selective transcripts in the prefrontal cortex: comparisons across schizophrenia and mood disorders. Psychological Medicine. 2024 Oct;54(14):3896–905.

44. Chung DW, Volk DW, Arion D, Zhang Y, Sampson AR, Lewis DA. Dysregulated ErbB4 Splicing in Schizophrenia: Selective Effects on Parvalbumin Expression. AJP. 2015 Sept 4;173(1):60–8.

45. Mellios N, Huang HS, Baker SP, Galdzicka M, Ginns E, Akbarian S. Molecular Determinants of Dysregulated GABAergic Gene Expression in the Prefrontal Cortex of Subjects with Schizophrenia. Biological Psychiatry. 2009 June 15;65(12):1006–14.
