# Supplementary material for: Cell Density and mRNA Expression of Inhibitory Interneurons in Schizophrenia: A Meta-Analysis
Source: bioRxiv. 2025 May 27:2025.05.23.655812. Preprint. [Version 1] doi: 10.1101/2025.05.23.655812 (PMC12154732; doi:10.1101/2025.05.23.655812)
Supplement: Supplementary file 1 [file media-2.pdf]

SUPPLEMENTARY MATERIAL

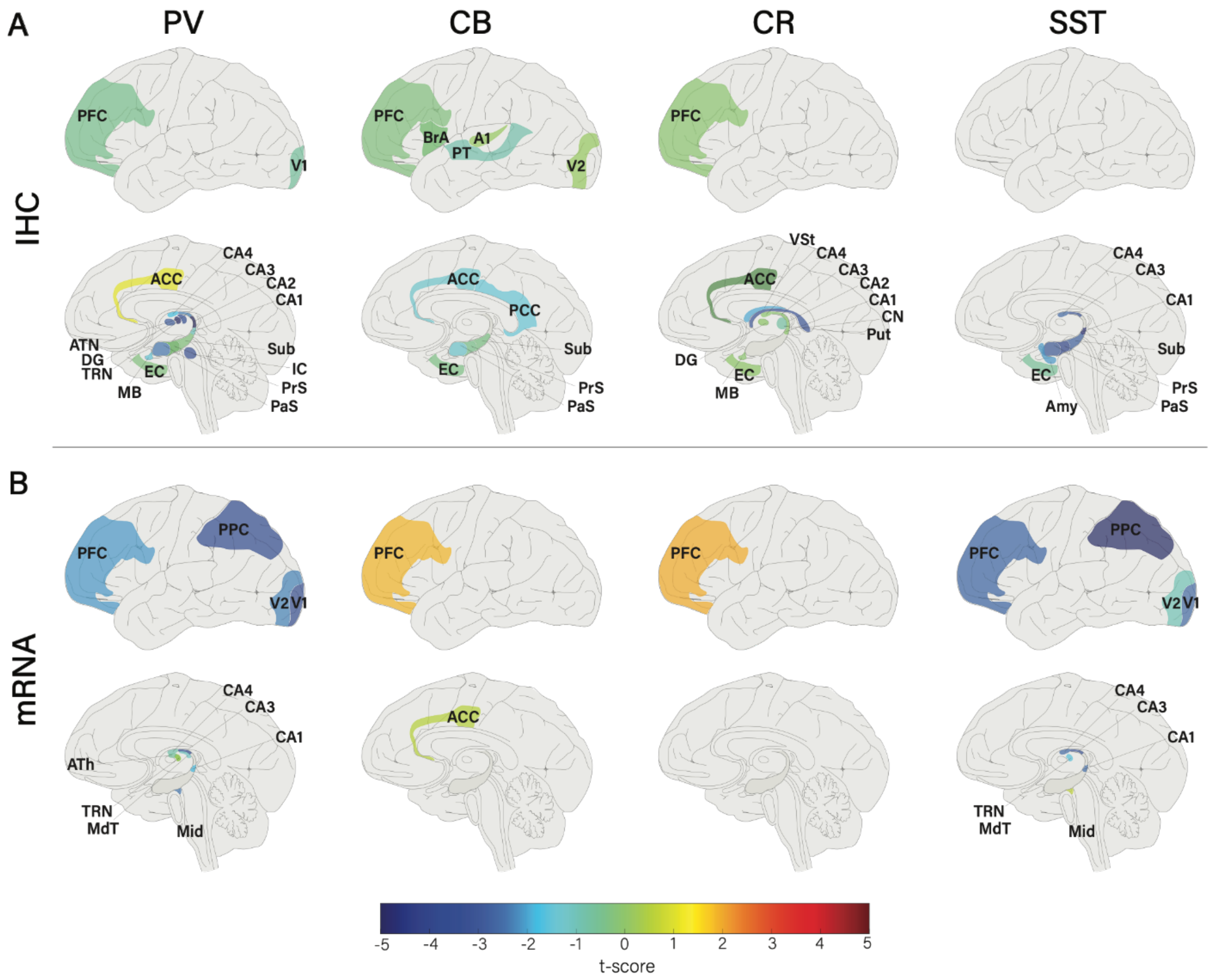

Supplementary Figure 1. Brain-wide alterations in interneuron density or expression for each cell type, method, and area. A) PV, CB, CR, and SST alterations measured by IHC (cell density). B) PV, CB, CR, and SST alterations in mRNA expression

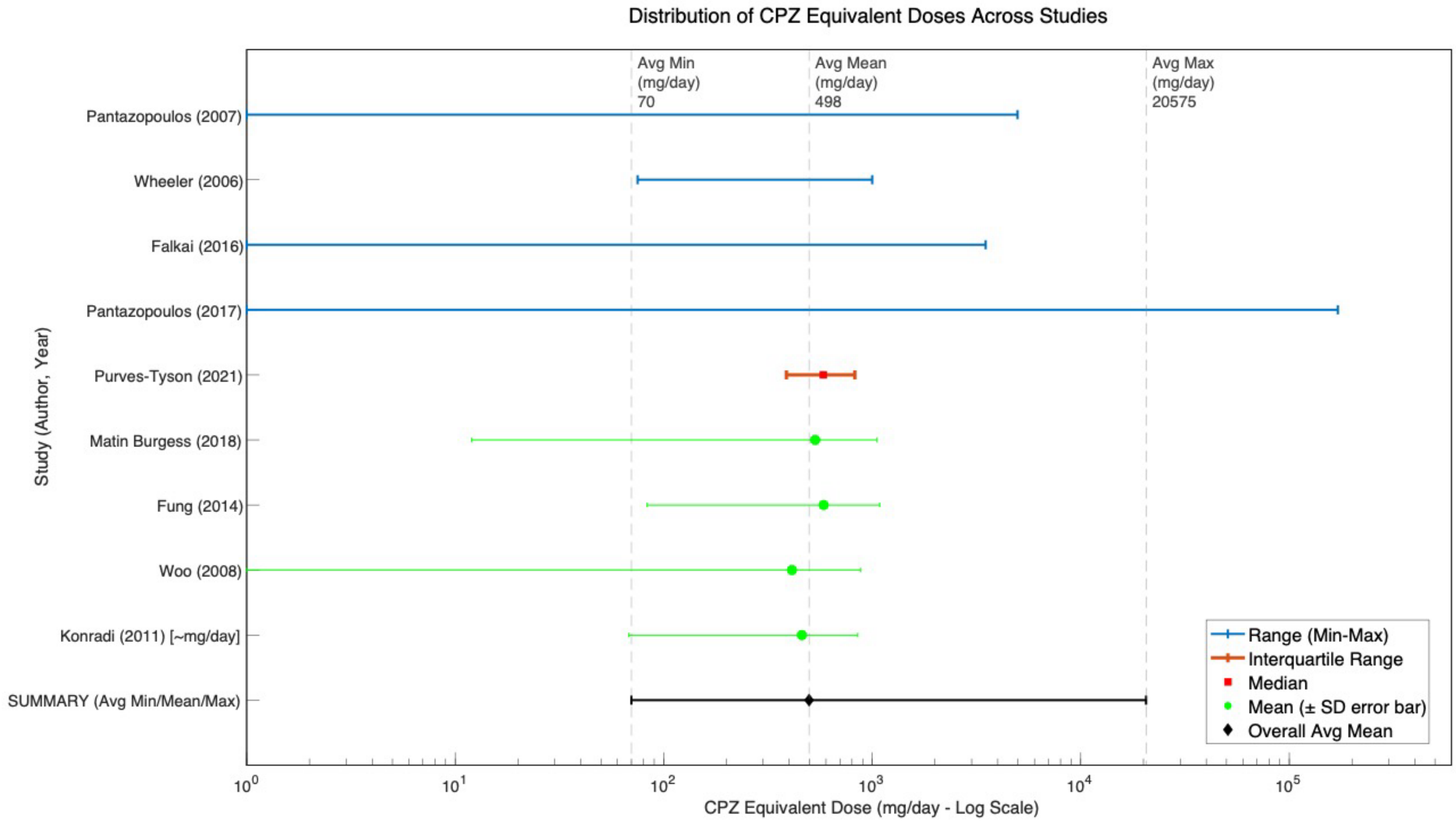

Supplementary Figure 2. Distribution of reported CPZ equivalent doses (mg/day) across 9 studies on a logarithmic scale. Markers indicate ranges (minimum/maximum values), means ± SD, or medians. The gray vertical lines and a black solid line show an overall average of the minimum, mean, and maximum values an studies that reported quantitative values.

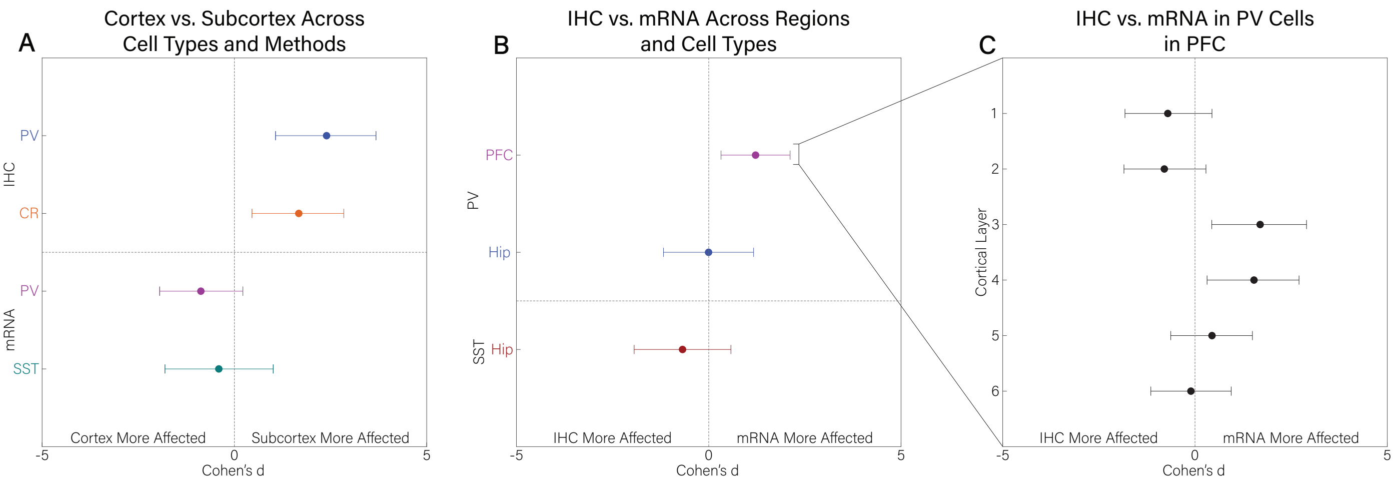

Supplementary Figure 3. Effect size analysis using *Cohen's d* on A) cortex versus subcortex in IHC and mRNA for PV, CR, and SST interneurons, B) IHC versus mRNA across brain regions for PV and SST interneurons, and C) IHC versus mRNA for each cortical layer in PV interneurons in the PFC.

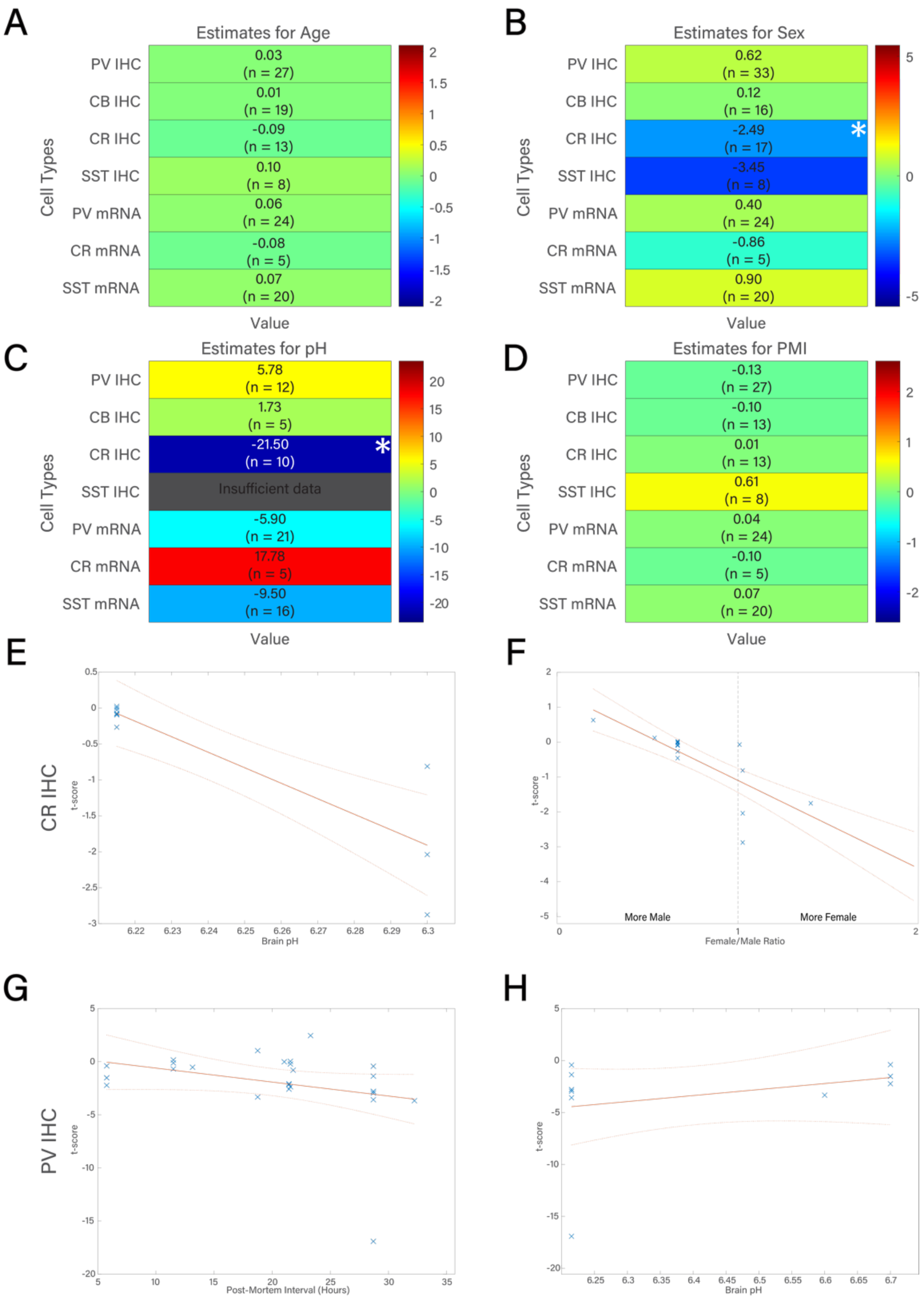

Supplementary Figure 4. (A-D) Regression coefficient estimates for meta-regression on weighted averaged A) age, B) sex, C) brain pH, and D) post-mortem interval. Significance determined by $p < 0.0018$ (Bonnferroni correction = 0.05 / 28). Number of observations (studies) included below the regression coefficient. Studies with insufficient regressor data ($n < 5$ observations/studies) were not included (CB mRNA - all regressors, SST IHC – brain pH). (E-H) Correlation analysis with a linear regression fit (red lines) and 95% confidence intervals (red dotted lines) show the relationship between mediating factors pH (CR IHC and PV IHC), sex (CR IHC), and post-mortem interval (PMI, PV IHC) and t-scores. PV IHC is plotted as an example displaying insignificance.

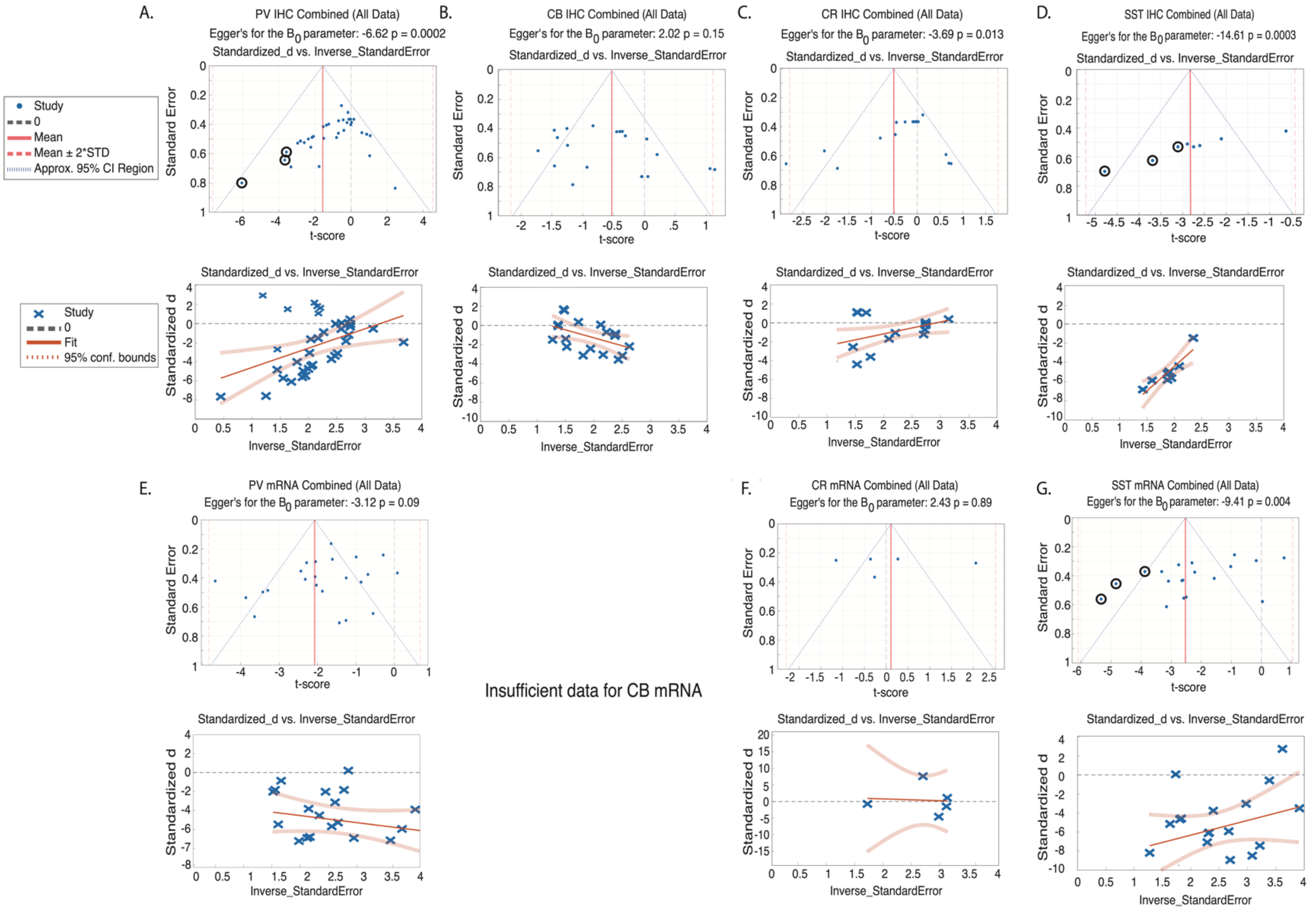

Supplementary Figure 5. Funnel plots (A-G) display the assessment of potential publication bias using Egger's test. For our analysis, we used a minimum of 5 studies. The x-axis represents the effect size (t-score), while the y-axis indicates standard error (SE). Black circles within the funnel plots highlight outlier studies that were removed to de-bias the distribution. The following studies were removed, but are not visible on the plot: PV IHC (A), one study (t-score = -16.9184, Author(s) = Zhang and Reynolds, Area(s) = CA2); CR IHC (C), one study (t-score = -3.4641, Author(s) = Adorjan, Area(s) = CN); SST mRNA (G), one study (t-score = -6.4371, Author(s) = Tsubomoto, Area(s) = PPC). The subplots below the Funnel plots display the regression analysis corresponding to the Egger's test. The x-axis represents precision (1/SE), and the y-axis represents the standardized effect size ($t'/SE(t')$). Also displayed are the linear regression fit (red lines) and 95% confidence intervals (red dotted lines).
